# Creatine and l-carnitine attenuate muscular laminopathy in the *LMNA* mutation transgenic zebrafish

**DOI:** 10.1038/s41598-024-63711-7

**Published:** 2024-06-04

**Authors:** Shao-Wei Pan, Horng-Dar Wang, He-Yun Hsiao, Po-Jui Hsu, Yung-Che Tseng, Wen-Chen Liang, Yuh-Jyh Jong, Chiou-Hwa Yuh

**Affiliations:** 1 Institute of Molecular and Genomic Medicine, National Health Research Institutes, Zhunan, Miaoli Taiwan; 2 Institute of Biotechnology, National Tsing Hua University, Hsinchu, Taiwan; 3 Department of Laboratory Medicine, Mackay Memorial Hospital, Taipei, Taiwan; 4 Department of Medical Laboratory Science and Biotechnology, Yuanpei University of Medical Technology, Hsinchu, Taiwan; 5 Department of Nursing, MacKay Medical College, Taipei, Taiwan; 6 Marine Research Station, Institute of Cellular and Organism Biology, Academia Sinica, I-Lan, Taiwan; 7 Department of Pediatrics, School of Medicine, College of Medicine, Kaohsiung Medical University, Kaohsiung, Taiwan; 8 Graduate Institute of Clinical Medicine, College of Medicine, Kaohsiung Medical University, Kaohsiung, Taiwan; 9 Department of Pediatrics, Kaohsiung Medical University Hospital, Kaohsiung Medical University, Kaohsiung, Taiwan; 10 Translational Research Center of Neuromuscular Diseases, Kaohsiung Medical University Hospital, Kaohsiung Medical University, Kaohsiung, Taiwan; 11 Department of Laboratory Medicine, Kaohsiung Medical University Hospital, Kaohsiung Medical University, Kaohsiung, Taiwan; 12 Drug Development and Value Creation Research Center, Kaohsiung Medical University, Kaohsiung, Taiwan; 13 Department of Biological Science and Technology, National Yang Ming Chiao Tung University, Hsinchu, Taiwan; 14 Institute of Bioinformatics and Structural Biology, National Tsing Hua University, Hsinchu, Taiwan; 15 Ph.D. Program in Environmental and Occupational Medicine, Kaohsiung Medical University, Kaohsiung, Taiwan

**Keywords:** Striated muscle laminopathy, Zebrafish, LMNA, Creatine, l-Carnitine, Drug discovery, Molecular medicine

## Abstract

*Lamin A/C* gene (*LMNA*) mutations contribute to severe striated muscle laminopathies, affecting cardiac and skeletal muscles, with limited treatment options. In this study, we delve into the investigations of five distinct *LMNA* mutations, including three novel variants and two pathogenic variants identified in patients with muscular laminopathy. Our approach employs zebrafish models to comprehensively study these variants. Transgenic zebrafish expressing wild-type *LMNA* and each mutation undergo extensive morphological profiling, swimming behavior assessments, muscle endurance evaluations, heartbeat measurement, and histopathological analysis of skeletal muscles. Additionally, these models serve as platform for focused drug screening. We explore the transcriptomic landscape through qPCR and RNAseq to unveil altered gene expression profiles in muscle tissues. Larvae of *LMNA*(L35P), *LMNA*(E358K), and *LMNA*(R453W) transgenic fish exhibit reduced swim speed compared to *LMNA*(WT) measured by DanioVision. All *LMNA* transgenic adult fish exhibit reduced swim speed compared to *LMNA*(WT) in T-maze. Moreover, all *LMNA* transgenic adult fish, except *LMNA*(E358K), display weaker muscle endurance than *LMNA*(WT) measured by swimming tunnel. Histochemical staining reveals decreased fiber size in all *LMNA* mutations transgenic fish, excluding *LMNA*(WT) fish. Interestingly, *LMNA*(A539V) and *LMNA*(E358K) exhibited elevated heartbeats. We recognize potential limitations with transgene overexpression and conducted association calculations to explore its effects on zebrafish phenotypes. Our results suggest lamin A/C overexpression may not directly impact mutant phenotypes, such as impaired swim speed, increased heart rates, or decreased muscle fiber diameter. Utilizing *LMNA* zebrafish models for drug screening, we identify l-carnitine treatment rescuing muscle endurance in *LMNA*(L35P) and creatine treatment reversing muscle endurance in *LMNA*(R453W) zebrafish models. Creatine activates AMPK and mTOR pathways, improving muscle endurance and swim speed in *LMNA*(R453W) fish. Transcriptomic profiling reveals upstream regulators and affected genes contributing to motor dysfunction, cardiac anomalies, and ion flux dysregulation in *LMNA* mutant transgenic fish. These findings faithfully mimic clinical manifestations of muscular laminopathies, including dysmorphism, early mortality, decreased fiber size, and muscle dysfunction in zebrafish. Furthermore, our drug screening results suggest l-carnitine and creatine treatments as potential rescuers of muscle endurance in *LMNA*(L35P) and *LMNA*(R453W) zebrafish models. Our study offers valuable insights into the future development of potential treatments for *LMNA*-related muscular laminopathy.

## Introduction

*LMNA* is a gene that encodes for nuclear lamins A and C, intermediate filament proteins of the nuclear lamina that are located at the inner nuclear membrane. The *LMNA* gene is composed of 12 exons, and alternative splicing in exon 10 produces two mRNAs that encode for pre-lamin A and lamin C. The lamin A/C protein is characterized by an N-terminal globular domain, a central α-helical coiled-coil rod domain, and a globular C-terminal tail domain^1^. Nuclear lamin A/C plays a crucial role in maintaining nuclear stability, regulating chromatin structure, and modulating gene expression, which occurs through the dynamic intermediate filament (IF) lamin filaments and A-type lamins that are located in the nucleoplasm^2^. In addition to its crucial role in maintaining nuclear structure, lamin A/C also participates in transcriptional regulation and DNA damage repair^3^. Lamin A/C proteins are important for the proper functioning of all organisms and have been highly conserved throughout evolution. To date, more than 498 different mutations have been identified in the human *LMNA* gene, the majority of which are single-point mutations^4^.

Mutations in the *LMNA* gene can cause a group of genetically and clinically heterogeneous disorders known as laminopathies. These disorders can be inherited in an autosomal dominant, autosomal recessive inheritance and affect various tissues, including striated muscle, nerve, adipose tissue, and skin. Examples of laminopathies include Emery–Dreifuss muscular dystrophy (EDMD), limb-girdle muscular dystrophy type 1B (LGMD1B) has been reclassified as EDMD2, congenital muscular dystrophy (CMD), dilated cardiomyopathy type 1A, familial partial lipodystrophy type 2, and early aging syndromes such as Hutchinson–Gilford progeria syndrome^5^. EDMD is primarily manifested with early childhood-onset limb weakness, muscle atrophy, contractures affect neck, elbow, and ankle, and adult-onset arrhythmia, increased risk of sudden cardiac death. In addition, studies using mouse models have suggested that *LMNA* mutations can also impair skeletal muscle growth^6^. The loss of lamin A/C in skeletal muscles has been linked to bone loss, potentially providing a pathological mechanism that links muscle and bone aging^7^.

Previous reports have suggested that *LMNA* mutations can cause nuclear envelope instability, resulting in deformation and rupture of the nuclear envelope when muscle cell nuclei move outside during muscle fiber development. This can lead to muscle cell apoptosis and dystrophin dysfunction^3^. The RNA-seq analysis of muscle biopsies from individuals with *LMNA* mutations showed upregulation of MAPK and AKT/mTOR pathways and downregulation of the AMPK pathway. These findings suggest that targeting the AMPK and mTOR pathways could be a potential therapeutic strategy for skeletal muscle laminopathies^8^. It has been reported that lamin A/C interacts directly with c-Fos at the nuclear envelope^9^. *LMNA* mutations can also lead to mitochondrial DNA replication defects, which may compromise the maintenance of the nuclear genome^10^.

Dr. Liang WC and Dr. Jong YJ provided care for 12 patients with muscular laminopathy from 5 families affected by CMD, EDMD2 and EDMD. DNA sequencing of patients’ sera identified five *LMNA* mutations, including three novel mutations. Specifically, a CMD patient exhibited *LMNA*(c.104T>C), resulting in an amino acid change from leucine to proline [p. Leu35Pro or *LMNA*(L35P)]^11^. An EDMD family, consisting of a grandfather, mother and daughter, displayed EDMD with *LMNA*(c.1616C>T), leading to an alanine-to-valine alteration [p.Ala539Val or *LMNA*(A539V)]^12^. Another EDMD family, comprising a mother and two daughters, showcased *LMNA*(c.1558T>G), causing tryptophan to become glycine [p.Trp520Gly or *LMNA*(W520G)]^13^.

Two pathogenic LMNA variants were identified as contributors to muscular laminopathies. Notably, two CMD sisters exhibited de novo *LMNA*(c.1072G>A) variant, resulting in a glutamic acid change to lysine [p.Glu358Lys or *LMNA*(E358K)]^14,15^. Additionally, an EDMD2 family, comprising a mother and two siblings, presented the *LMNA*(c.1357C>T) variants, causing an arginine-to-tryptophan alteration [p.Arg453Trp or *LMNA*(R453W)]^16,17^. To maintain a consistent writing format for protein variants, we will adopt the shorter format throughout the entire manuscript.

While management primarily focuses on providing support and addressing symptoms, including the necessity of pacemaker implantation for severe conduction defects and bradycardia, there is currently no cure for patients with muscular laminopathy^18^. Table 1 summarizes the patient phenotypes associated with each lamin A/C variant under investigation. In this study, we employed zebrafish for both functional and histopathological assessments, establishing a disease platform for therapeutic screening. Our analysis confirmed that all the affected amino acids in our study are conserved in the zebrafish lamin A/C protein (Fig. S1). Sequence alignment were performed using Multiple sequence alignment by Florence Corpet^19^.

**Table 1:** Phenotype and genotype of patients with muscular laminopathy.

Family	LMNA mutation	Pathogenicity of variant	Genotype/protein domain	Phenotype	Members	References
1	Missense	Novel	c.104T>C (L35P)/Coil 1A	CMD	Adopted child	^11^
2	Missense	Novel	c.1616C>T (A539V)/Ig-fold	AD-EDMD	Grandfather, mother and daughter	^12^
3	Missense	Novel	c.1558T>G (W520G)/Ig-fold	AD-EDMD	Mother and 2 daughters	^13^
4	Missense	Pathogenic	c.1072G>A (E358K)/Coil 2B	CMD	Two sisters	^14,15^
5	Missense	Pathogenic	c.1357C>T (R453W)/Ig-fold	LGMD-1B	Mother and 2 siblings	^16,17^

*CMD* congenital muscular dystrophy, *AD-EDMD* autosomal dominant Emery–Dreifuss muscular dystrophy, *LGMD-1B* limb-girdle muscular dystrophy type 1B, *Ig-fold* Immunoglobulin fold.

Zebrafish (*Danio rerio*) is an invaluable model organism for studying vertebrate development and genetics, offering advantages such as rapid development, high reproductive capacity, advanced genetic modification tools, and transparency of embryos for internal morphology observation^20^. With significant genetic similarities to humans and the ability to easily modulate gene expression, zebrafish has emerged as an ideal model for studying human diseases in various fields such as ALS^21^, cancer biology and precision medicine^22^, anxiety^23^, and neurodegenerative disease^24^. Specifically, zebrafish’s suitability for investigating muscular dystrophy stems from its easily mutating disease-causing genes, imaging intact muscle tissue, and using for therapeutic screening^25^.

Zebrafish is an excellent model for studying somite muscle development due to its rapid and segmented paraxial mesoderm. Slow-twitch fibers are the first to differentiate at 16 h post-fertilization (hpf) and migrate to the lateral periphery of the somites, followed by the rapid development of a large quantity of fast-twitch muscle fibers^26^. Muscle differentiation in zebrafish is complete at 48 hpf, with the vertical myoseptum partitioning the somites into fast and slow muscle fibers, similar to the myotendinous junctions (MTJ) in mammals^27^. Our previous study established transgenic fish expressing *tropomyosin 3* (*TPM3*) mutations and demonstrated that *TPM3*(E151G) and *TPM3*(E151A) exhibit different pathogenicity. Furthermore, we identified l-Carnitine as a supplement that can ameliorate muscle endurance weakness in transgenic fish with *TPM3* mutations^28^. This proof-of-concept study highlights the potential of zebrafish as a perfect model for congenital myopathy and drug screening.

In this study, we aimed to investigate the relationship between *LMNA* mutations and muscular laminopathy by establishing six different *LMNA* transgenic zebrafish lines. To generate the transgenic lines, we used the skeletal muscle-specific *myosin light chain 2* (*mlc2*) promoter to express the wild-type (WT) human *LMNA* as well as the five different *LMNA* mutations, while the heart-specific *myosin light chain 7* (*myl7*) promoter was used to drive the expression of the *EGFP* transgene as a reporter. Our control group was *Tg (mlc2:LMNA(WT); myl7:EGFP)*, which expresses the wild-type *LMNA* gene. We created *Tg (mlc2:LMNA(L35P); myl7:EGFP)*, *Tg (mlc2:LMNA(A539V*); myl7:EGFP), and *Tg (mlc2:LMNA(W520G); myl7:EGFP)* by using the *mlc2* promoter to drive the expression of the three different *LMNA* mutations, which are novel and derived from the patients. In addition, we also established two other transgenic fish lines, *Tg (mlc2:LMNA(E358K); myl7:EGFP)* and *Tg (mlc2:LMNA(R453W); myl7:EGFP)*, which carried pathogenic *LMNA* variants that had been previously isolated and reported to cause EDMD^29^. Our results revealed those novel *LMNA* variants in the transgenic zebrafish result in muscle dysfunction and decreased fiber size, similar to human laminopathies. l-carnitine and creatine treatments demonstrate improved muscle endurance for specific *LMNA* mutants (L35P and R453W), offering potential therapeutic options for muscular laminopathy.

## Materials and methods

### Zebrafish maintenance

The zebrafish used in this study were maintained at the Taiwan Zebrafish Core Facility (TZCF), which operates under an automatic 14/10-h light/dark cycle and maintains the water temperature at 28 °C by using a circulating water system. Adult fish were normally fed twice daily at 10 a.m. and 3 p.m. unless specifically mentioned. This study was approved by the Ethics Committee: Institutional Animal Care and Use Committee (IACUC) of National Health Research Institutes, with ethics approval reference NHRI-IACUC-111015-A. All methods were carried out in accordance with relevant guidelines and regulations. We confirm that all methods are reported in accordance with ARRIVE guidelines. TZCF is accredited by AAALAC International, with a full certification obtained in 2015.

### Transgenic zebrafish lines

AB(WT) was used in this study. The other transgenic fish lines, including *Tg(mlc2:LMNA(WT);myl7:EGFP)*, *Tg(mlc2:LMNA(L35P);myl7:EGFP)*, *Tg(mlc2:LMNA(E358K);myl7:EGFP)*, *Tg(mlc2:LMNA(R453W);myl7:EGFP)*, *Tg(mlc2:LMNA(W520G);myl7:EGFP)*, and *Tg(mlc2:LMNA(A539V);myl7:EGFP)*, were generated by using the Tol2 Gateway cloning Toolkit as described previously^30,31^. The primers used for the construct preparation are listed in Table S1.

Expression vectors were constructed using the multisite Gateway® Three-Fragment Vector Construction Kit as described earlier^28^. LMNA cDNA was amplified by PCR using the attB-LMNA-F and attB-LMNA-R primers (Table S1), and combined with the pDONR221 vector to generate the middle entry clone (pME-*LMNA*(WT)) through the BP reaction. The resulting DNA products were purified, and the entry clones were sequenced using the primers listed in Table S1. The LR reaction was employed to generate the final expression construct (*pTol2-mlc2:LMNA(WT):pA/CG*), and correct colonies were selected after colony PCR and plasmid PCR. Site-directed mutagenesis was performed using the QuickChange II kit and specific primers (listed in Table S2) to create five different *LMNA* variants, and the mutant sites were confirmed by sequencing before proceeding with the LR reaction.

### Microinjection and selection and confirmation of transgenic zebrafish

To efficiently excise and integrate the DNA fragment surrounded by the Tol2 element transposon into the genome, we employed co-injection of in vitro-transcribed Tol2 transposase mRNA as described earlier^28^. The injected embryos were then placed in a Petri dish with E3 medium and kept in a 28 °C incubator. At 3 days post-fertilization (dpf), we screened for larva with green fluorescence in the heart under a microscope derived from the *myl7*:EGFP reporter. The embryos that showed EGFP expression were raised to adulthood, and the genomic DNAs were extracted from their fin clips using a method involving NaOH (50 mM) and heating at 95 °C. Subsequently, we performed PCR and DNA sequencing to verify the gene sequence, using the primers listed in Table S1, and confirmed that the sequences are correct and the constructs exist in the transgenic lines. In our observations, we noted that the proportions of different genotypes among the offspring closely adhered to the expected Mendelian ratios within acceptable margins. Specifically, approximately 50% of the offspring were heterogeneous after fin clip genomic identification, while 50% were wild-type, aligning with classical Mendelian inheritance patterns.

### Fish length and body weight measurement and morphological analysis

Prior to examination, the fish were anesthetized using 0.016% tricaine buffer (ethyl 3-aminobenzoate methanesulfonate acid salt, Sigma-Aldrich, USA). We measured the total length and standard length of each fish using Vernier calipers with 0.1 mm precision, and recorded their body weight with an accuracy of 0.01 g. For morphological analysis of the vertebrae, we used a digital CMOS X-ray detector (Model 2315, Dexela, London, UK) at 45 kV and 120 mA, with a 2.5 s exposure time^32^.

### Tracking of fish larvae swim speed

We monitored the swim speed of fish larvae using the DanioVision system (EthoVision XT14, Noldus). Each larva was placed in a separate well of a 48-well plate containing 1000 µl of E3 buffer and kept at 28 °C in a dark room to adapt for 30 min. Then, we exposed the larvae to alternating light and dark conditions for two cycles, with 10 min of light on followed by 10 min of light off. We used the EthoVision XT 14 software (Noldus, The Netherlands, https://www.noldus.com/ethovision-xt) to automatically track and measure the velocity of each larva in the experiment.

### T-maze behavior test for adult fish swimming speed

We used a T-maze behavior test to measure the swim speed of adult fish. The test consisted of pre-training, training, and a test phase, where fish were trained to find food on a sponge in the left arm of the maze, as described in our previous paper^28^. Their swim behavior was recorded, and swim speed was analyzed using EthoVision XT14 software.

### Swimming tunnel for adult fish muscle endurance

To investigate the relationship between *LMNA* variants and muscular laminopathy, we assessed the muscle endurance of adult transgenic fish using a Brett-type intermittent-closed swimming tunnel (38 cm long, 10 cm wide and 9.5 cm deep; Loligo Systems ApS, Tjele, Denmark) at the Marine Research Station. After a 1-min habituation period, the fish were subjected to water flow resistance, and their *U*_crit_ (critical swimming speed, represented as cm/s) was calculated using a formula based on maximum velocity and time to fatigue as described earlier^28^.

### Chemical treatment for larvae fish

To identify potential drugs for the therapy of muscular laminopathy, we selected five muscle-improving drugs for treatment of the larvae: l-tyrosine (10 µM in water), Taurine (1 mM in water), l-carnitine (10 µM in water), creatine (100 µM in water), and Terazosin (2.5 µM in diluted DMSO). The concentrations of those drugs were determined based on the previous studies^28,33,34^. We placed the larvae fish in Petri dishes and administered the different drugs at 28 hpf. The larvae fish were maintained in a 28 °C incubator and their swim speed was tested via DanioVision for 5 dpf to 8 dpf.

### Chemical treatment for larvae fish up to 1 month

To further investigate the effects of drug treatment on the fish, we selected the drugs that showed an increase in swim speed in larvae and continued treatment for 1 month. The drug treatment was initiated at 28 hpf, and after allowing the larvae to immerse throughout the day until 7 dpf, we transferred them to a 1-l fish tank. At 8 am, we changed 800 ml of water and fed the fish four times a day (at 9 am, 11 am, 1 pm, and 4 pm), followed by a 12-h (from 8 pm to 8 am) treatment period in a Petri dish with the same drug concentration as the larvae fish treatment. This treatment and feeding regimen were repeated daily until 30 dpf.

### Tissue collection for molecular analysis

To conduct molecular analysis, zebrafish skeletal muscle was used. For the 7-dpf larvae, all fish were collected and frozen at − 80 °C for RT-qPCR. For adult fish, the head and fin were removed, and the organs were hollowed out. The body was then divided into the anterior and posterior parts. The anterior part was frozen at − 80 °C for RT-qPCR analysis, and the posterior part was used for sample preparation and frozen section.

### RNA extraction and reverse transcription-quantitative polymerase chain reaction (RT-qPCR)

We isolated RNA from zebrafish skeletal muscle using the NucleoSpin® RNA Midi kit (MACHEREY–NAGEL, US), and performed reverse transcription-quantitative PCR (RT-qPCR) according to the protocol as described in our paper^28^. Briefly, each sample underwent homogenization in a mixture of 350 µl RA1 Buffer, 3.5 µl β-Mercaptoethanol, and magnetic beads. The lysate was processed through NucleoSpin® filter columns, followed by ethanol addition, desalting, and DNase digestion. RNA concentration was determined using a Nanodrop ND-1000 UV–Vis spectrophotometer, and samples were stored at − 80 °C.

For cDNA synthesis and qPCR, the iScript™ cDNA synthesis kit (Bio-Rad, US) was employed. The reaction mixture, consisting of 1 µg RNA template, 1 µl iScript Reverse Transcriptase, 4 µl 5× iScript Reaction Mix, and Nuclease-free water, was subjected to priming at 25 °C, reverse transcription at 46 °C, and inactivation at 95 °C. The resulting cDNA was diluted 100 folds, and 1.2 µl primer and 5 µl 2X SYBR Green were mixed before loading into a 384-well plate. Initial loading of 3.8 µl cDNA was followed by the addition of the 2× SYBR Green and primer mixture. qPCR primers from Table S1 were employed, and the program included a 40-cycle PCR stage and subsequent melt curve stage. Triplicate qPCR analyses were conducted for each sample to minimize technical errors.

### Preparation of the muscle specimens and frozen section

To prepare frozen muscle sections, we first anesthetized adult fish with a 0.025% buffered MS-222 solution (Sigma-Aldrich, US), and then removed a segment of muscle tissue located between the anal and caudal fins^35^. We next mixed tragacanth gum powder (Wako, Japan) with water to form a homogenous blend, and placed it on a wooden surface in the shape of a small hill. We divided the posterior part of the muscle tissue into two sections and fixed them onto the small hill. Next, we filled a beaker with 2-methylbutane (Sigma-Aldrich, US) and placed it in a barrel of liquid nitrogen. Once the white smoke had dissipated, we immersed the muscle sample together with the wooden support in the 2-methylbutane for 1 min, and transferred it immediately to a − 80 °C freezer. We then used a Cryostat Microtome CM3050S (Leica, Germany) to section the muscle sample, the tissue sections were stored at − 80 °C for later Hematoxylin and Eosin stain (H&E stain), Gömöri trichrome stain, and nicotinamide adenine dinucleotide dehydrogenase‐tetrazolium reductase (NADH-TR) stain as described previously^28^.

### Analysis of next generation sequencing data

The next generation sequencing (NGS) experiment was conducted by Taiwan Genome Industry Alliance Inc. as described previously^28^. The entire RNA sample preparation procedures were executed following the official protocols set by Illumina.

The parameters employed for the expression analysis were as follows: Genes with a fold change of less than − 2 or greater than 2 were considered, along with a gene-level P-value threshold of less than 0.05. The ANOVA method, specifically the Ebayes approach, was utilized for this analysis. Additionally, gene ontology analysis was conducted through the Ingenuity Pathway Analysis (IPA, Qiagen, Hilden, Germany) to create figures of the enriched canonical pathways. The NGS data have been submitted to the NCBI Gene Expression Omnibus (GEO) under accession code GSE242251.

### Statistical analysis

The data were analyzed by using GraphPad Prism software (version 10.2.0, https://www.graphpad.com/) and statistical analysis with either the two-tailed Student’s t-test or one-way ANOVA as described in the figure legends. To address the issue of multiple comparisons, we employed one-way ANOVA and comparing the mean of each column with the mean of control column LMNA(WT), or no drug control, therefore, we corrected for multiple comparisons using Dunnett’s test. The level of significance is shown by using asterisks: ns for not significant, *for 0.01 < P ≤ 0.05, **for 0.001 < P ≤ 0.01, ***for 0.0001 < P ≤ 0.001, and ****for P ≤ 0.0001. To evaluate the potential impact of lamin A/C overexpression on the observed phenotypes, we employed association calculations through correlation matrix analysis using Prism software. In a correlation matrix, each row and column represents the variables of interest, creating a square matrix that shows the correlation coefficients between all pairs of variables. The values in the matrix indicate the strength and direction of the linear relationship between the variables. Expression levels of lamin A/C in transgenic fish were imported alongside data pertaining to swim speed, muscle endurance, heart rates, and muscle fiber diameter. Each sample was represented by a row in the dataset, with columns corresponding to the variables under investigation. In pursuit of elucidating the relationship between gene expression and phenotype severity, Pearson correlation analysis was selected to quantify the strength and direction of this association.

## Results

### Establishing transgenic zebrafish models for *LMNA*-related muscular laminopathy

To investigate the pathogenesis of *LMNA*-related muscular laminopathy, we created transgenic zebrafish lines expressing human wild-type *LMNA* and five *LMNA* mutants. These transgenic lines were generated by utilizing the *myosin light chain 2* promoter (*mlc2*) to drive the expression of different *LMNA* cDNA, while a heart-specific *myosin light chain 7* (*my17*) promoter to drive the expression of enhanced green fluorescent protein (EGFP) was used as a reporter. The specific mutations corresponding to patients’ DNAs were introduced by using site-directed mutagenesis. The final constructs resulting from the LR reaction were confirmed by DNA sequencing. The validated expression constructs were microinjected into zebrafish embryos of the AB(WT) strain. At 3 dpf, the transgenic zebrafish larvae were selected based on the heart-specific green fluorescence. These embryos were raised until 3 months of age (referred to as F0 founders) and then mated with wild-type zebrafish to generate F1 transgenic fish. Self-crossing was performed to produce F2 transgenic fish. In total, we generated six *LMNA* transgenic zebrafish lines: *LMNA*(WT), 3 novel and 2 known pathogenic mutant lines, including *LMNA*(L35P), *LMNA*(E358K), *LMNA*(R453W), *LMNA*(W520G), and *LMNA*(A539V), and which were validated by fin-clip, and the presence of the intended mutations was confirmed by comparing them to the *LMNA*(WT) sequence (Fig. S2).

### Abnormal phenotypes observed in *LMNA*(A539V) and *LMNA*(R453W) transgenic zebrafish

We accomplished the successful integration of these constructs into zebrafish embryos, followed by the identification of larvae carrying transgenes through fluorescence microscopy examination on 3 dpf. The selected larvae were then nurtured until reaching adulthood. To pinpoint transgenic fish bearing specific *LMNA* mutations, fin clipping was employed. The majority of the fish, excluding *LMNA*(W520G), matured into adulthood and were subsequently crossbred with wild-type (WT) fish to produce F1 offspring. Notably, the *LMNA*(W520G) protein variant displayed a distinctive and unprecedented trait. Unlike other transgenic lines we established, where viable F0 adult fish were obtainable, the F0 fish carrying the *LMNA*(W520G) variant did not survive beyond the F0 generation. Despite appearing to swim as well as controls, their offspring die prematurely, leading to the unavailability of F1 fish for our analysis. This phenomenon of premature mortality is exceptional and has not been observed in our other transgenic models. The untimely demise of these fish is an intriguing characteristic, especially considering its relevance to the muscular laminopathy pathology. Lmna(ΔK32) knock-in mouse model exhibited incomplete tissue maturation and severe metabolic defects, ultimately leading to premature death^36^. Drawing from this parallel, we hypothesize that the premature death observed in our *LMNA*(W520G) fish may indeed reflect the pathogenicity of the protein variant.

**Figure 1:**
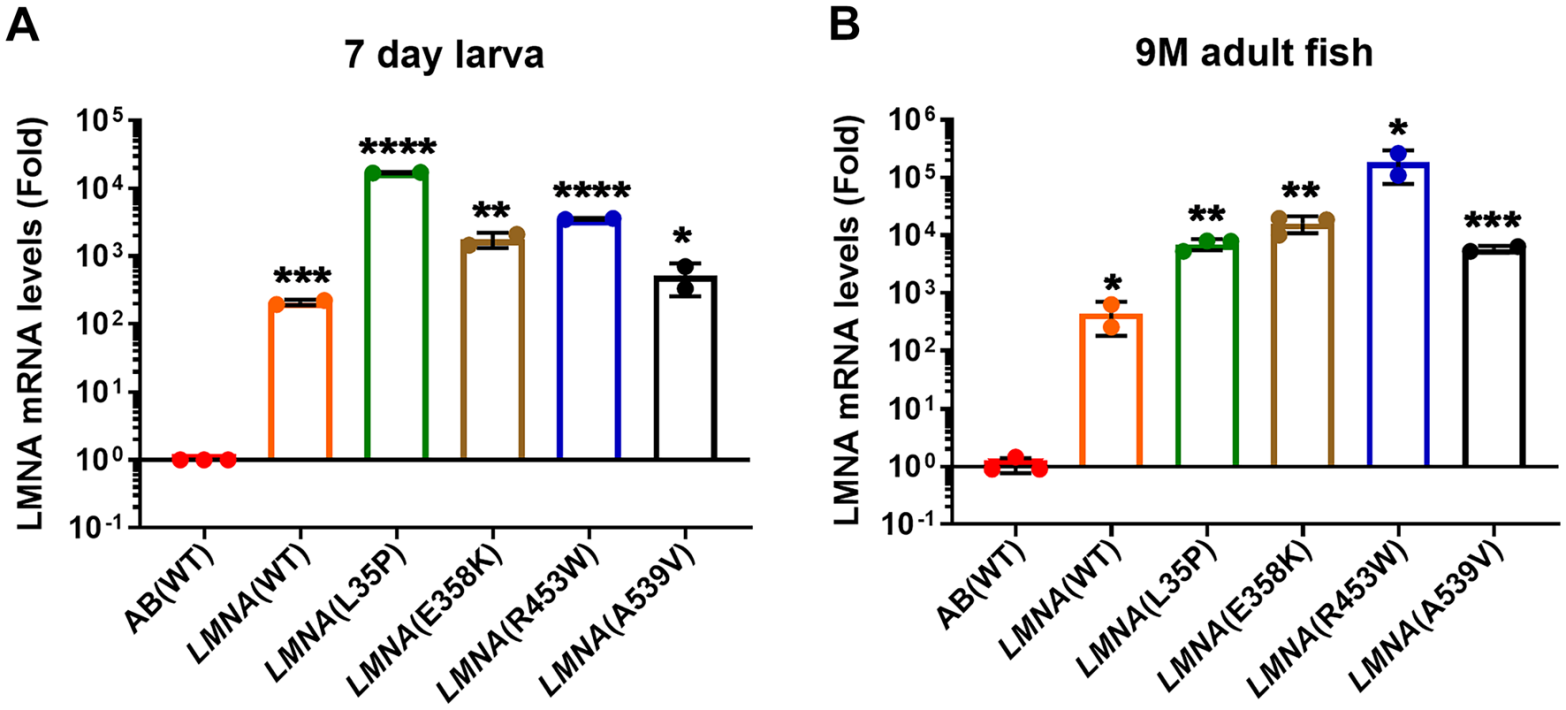
Elevated *LMNA* mRNA levels in the *LMNA* transgenic zebrafish. *LMNA* mRNA expression levels in (**A**) 7-day-old F1 larvae (N = 20 larvae), each dot represents the mRNA extracted from 20 larva. (**B**) 9-month-old F1 adult of AB(WT) and various *LMNA* transgenic fish (N = 3 adult fish), each dot represents the mRNA extracted from one adult fish. Statistical analysis was performed using ordinary One-way ANOVA with Dunnett’s test, and the level of statistical significance is indicated by the following notation: *0.01 < P ≤ 0.05; **0.001 < P ≤ 0.01; ***0.0001 < P ≤ 0.001; ****P ≤ 0.0001.

To confirm the expression of *LMNA* in the transgenic fish, RT-qPCR was conducted by using samples obtained from 7-dpf larvae and 9-month-old F1 adult transgenic fish. Compared to the control AB(WT) fish, the *LMNA*(WT) and the *LMNA* mutant transgenic fish (except *LMNA*(W520G) because no fish survived after F0) showed significantly higher levels of *LMNA* mRNA expression (Fig. 1A,B). The variability in the expression levels of human lamin A/C in larvae may be attributed to the larva being F0, introducing heterogeneity. Additionally, the observed fluctuations in expression levels across different life stages could stem from the dynamic nature of developmental processes and the regulatory mechanisms governing gene expression. Such variations are not uncommon and have been reported in previous studies^37–39^.

Subsequently, we proceeded to characterize the phenotypes and mobility of these transgenic fish. We observed some abnormalities in certain *LMNA* transgenic fish. While all *LMNA*(WT) fish exhibited a normal phenotype (Fig. S3A1), the 4-month-old F0 *LMNA*(A539V) fish displayed abnormal appearances: 2 out of 7 fish (29%) had no tail (Fig. S3A2,A2ʹ), and 3 out of 7 fish (43%) exhibited facial deformity (protruding jaw) (Fig. S3A3,A3ʹ). On the other hand, the other F0 *LMNA* variant fish lines appeared normal phenotype like LMNA(WT) fish.

In our study, we initially generated multiple lines for five LMNA mutants and observed similar larval phenotypes and LMNA expression levels across these lines. Based on these consistent findings, we selected a representative line with higher LMNA expression for each mutant for further experiments. This approach aligns with established zebrafish research practices and allows for manageable and focused experimentation, considering the impracticality of utilizing all possible lines in our study.

We crossed the *LMNA*(A539V) F0 fish with WT fish to observe whether these traits can be inherited. Once again, all F1 offspring of the *LMNA*(WT) fish displayed a normal phenotype (Fig. S3B1). However, the *LMNA*(A539V) fish without tails were unable to produce viable offspring and died at 4 months. The offspring of the *LMNA*(A539V) fish with protruding jaw did not exhibit the same traits, but some of the fish displayed a severe crooked body phenotype (Fig. S3B2). Similarly, the *LMNA*(R453W) F1 fish also exhibited a crooked body phenotype (Fig. S3B3). The remaining *LMNA* variant fish except *LMNA*(W520G), both in the F0 and F1 generations, appeared normal. *LMNA*(A539V) and *LMNA*(W520G) were considered novel protein variants at the time of our study’s inception, and based on the phenotypes observed in the F0 and F1 transgenic fish, we speculate that both variants may possess pathogenic roles.

### Slower swim speed in transgenic larvae carrying pathogenic *LMNA* mutations

To assess the impact of *LMNA* variants on muscle development and swim ability, we measured the swim speed of transgenic larvae using DanioVision (Fig. S4A–C). At 6 dpf, the *LMNA*(WT) F1 larvae exhibited slightly slower swim speed than AB(WT). However, the transgenic F1 larvae carrying *LMNA*(E358K), *LMNA*(R453W), and *LMNA*(A539V) variants exhibited significantly slower swim speed compared to the *LMNA*(WT) (Fig. S4A). At 7 dpf, *LMNA*(E358K) and *LMNA*(R453W) mutants F1 larvae displayed reduced swim speeds than *LMNA*(WT) (Fig. S4B). Furthermore, at 8 dpf, all *LMNA* mutants showed decreased swim speed compared to *LMNA*(WT) except *LMNA*(E358K) which only had four larvae left (Fig. S4C, Table 2). The pathogenic roles of *LMNA*(E358K) and *LMNA*(R453W) were previously reported to cause muscular laminopathy including CMD, EDMD, and EDMD2, our results confirm the pathological roles of *LMNA*(E358K) and *LMNA*(R453W) variants and suggest that the novel variants *LMNA*(L35P) and *LMNA*(A539V) may also have pathogenic implications.

**Table 2:** Pathogenicity of LMNA variants, phenotypes, swimming speed and muscle endurance of the LMNA variants transgenic fish.

Pathogenicity	Variants	Phenotype/survival	F1 larva swimming speed (DanioVision)	F1 adult swimming speed (T maze)	F1 adult muscle endurance (swimming tunnel)	Heartbeat	Muscle fiber Size
Novel	L35P	Normal	Slower swim speed	Slower swim speed	Weaker muscle strength	Normal	Reduced muscle fiber
Novel	A539V	Crooked body	Slower swim speed	Slower swim speed	Weaker muscle strength	Elevated heartbeat	Reduced muscle fiber
Novel	W520G	Premature death	–	–	–	–	–
Pathogenic	E358K	Normal	Slower swim speed	Slower swim speed	Weaker but not significant muscle strength	Elevated heartbeat	Reduced muscle fiber
Pathogenic	R453W	crooked body	Slower swim speed	Slower swim speed	Weaker muscle strength	Normal	Reduced muscle fiber

### Impaired swimming speed in adult transgenic fish with *LMNA* mutations

To investigate the effect of *LMNA* variants on the locomotion of adult transgenic fish, we conducted T-maze experiments to measure swim speed. The fish underwent a 1-week pre-training period during which they were fed with food placed in a sponge. In the training phase, the fish swam from the start box to the sponge located in the left arm of the T-maze, where food was placed. After 1 week of training, the fish were tested, and their swimming velocities were assessed.

Initially, during the early day 2 of the testing phase, no significant differences in swim speed were observed (Fig. S4D). Intriguingly, on the day 3, we observed slower swim speed in F0 transgenic fish carrying the *LMNA*(L35P), *LMNA*(R453W), *LMNA*(W520G), and *LMNA*(A539V) variants compared to *LMNA*(WT) fish (Fig. S4E). This trend continued on the day 4, with *LMNA*(L35P), *LMNA*(R453W), and *LMNA*(A539V) transgenic fish consistently exhibiting slower swim speed than *LMNA*(WT) fish (Fig. S4F). Despite the heterogeneity of the F0 fish, their reduced swim speed still indicated the pathogenic nature of the *LMNA* mutants, which is a significant finding.

**Figure 2:**
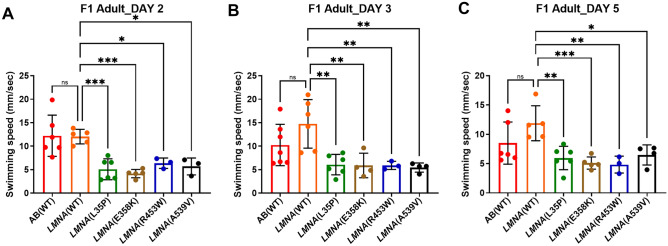
Swim speed analysis of F1 adult zebrafish expressing mutant human lamin A/C with those expressing WT human lamin A/C. (**A–C**) Swim speed analysis of F1 adult fish using T-maze behavior. The red plot represents AB(WT) fish, orange plot represents *LMNA*(WT) fish, green plot represents *LMNA*(L35P) fish, brown plot for *LMNA*(E358K) fish, blue plot for *LMNA*(R453W) fish, purple plot for *LMNA*(W520G) fish, and black plot for *LMNA*(A539V) fish. Each dot represents one fish. AB(WT): n = 6; *LMNA*(WT): n = 6; *LMNA*(L35P): n = 6; *LMNA*(E358K): n = 5; *LMNA*(R453W): n = 3; *LMNA*(A539V): n = 4. Statistical analysis was performed using ordinary One-way ANOVA comparing the mean of each column with the mean of control column (*LMNA*(WT)), and corrected for multiple comparisons using Dunnett’s test. The presented p-values have been appropriately adjusted, and the level of statistical significance is indicated by the following notation: *0.01 < P ≤ 0.05; **0.001 < P ≤ 0.01; ***0.0001 < P ≤ 0.001; ****P ≤ 0.0001.

Due to the mosaic nature of expression of the transgene in the F0 generation, we further evaluated the swim speed of F1 adult fish. We compared zebrafish expressing WT human lamin A/C with non-transgenic WT fish and zebrafish expressing mutant human lamin A/C with those expressing WT human lamin A/C. While the swim speed of *LMNA*(WT) F1 adults did not significantly differ from that of the AB(WT) fish, *LMNA*(L35P), *LMNA*(E358K), *LMNA*(R453W) and *LMNA*(A539V) fish displayed significantly slower swim speed from day 2 to day 5 compared to *LMNA*(WT) fish (Fig. 2, Table 2). In conclusion, our findings disclose that the transgenic fish carrying *LMNA* variants exhibit slower swim speed both in the larval and adult stages. The reduced swim speed observed in both F0 and F1 adults with *LMNA*(E358K) and *LMNA*(R453W) transgenic fish are consistent with their previously reported pathogenic roles, which validates our findings in the other *LMNA* variants. Furthermore, the novel *LMNA*(L35P) and *LMNA*(A539V) variants lead to slower swim speed in both F0 and F1 adult fish, providing evidence of their pathogenic nature.

### Decreased muscle endurance in adult transgenic fish with *LMNA* mutations

**Figure 3:**
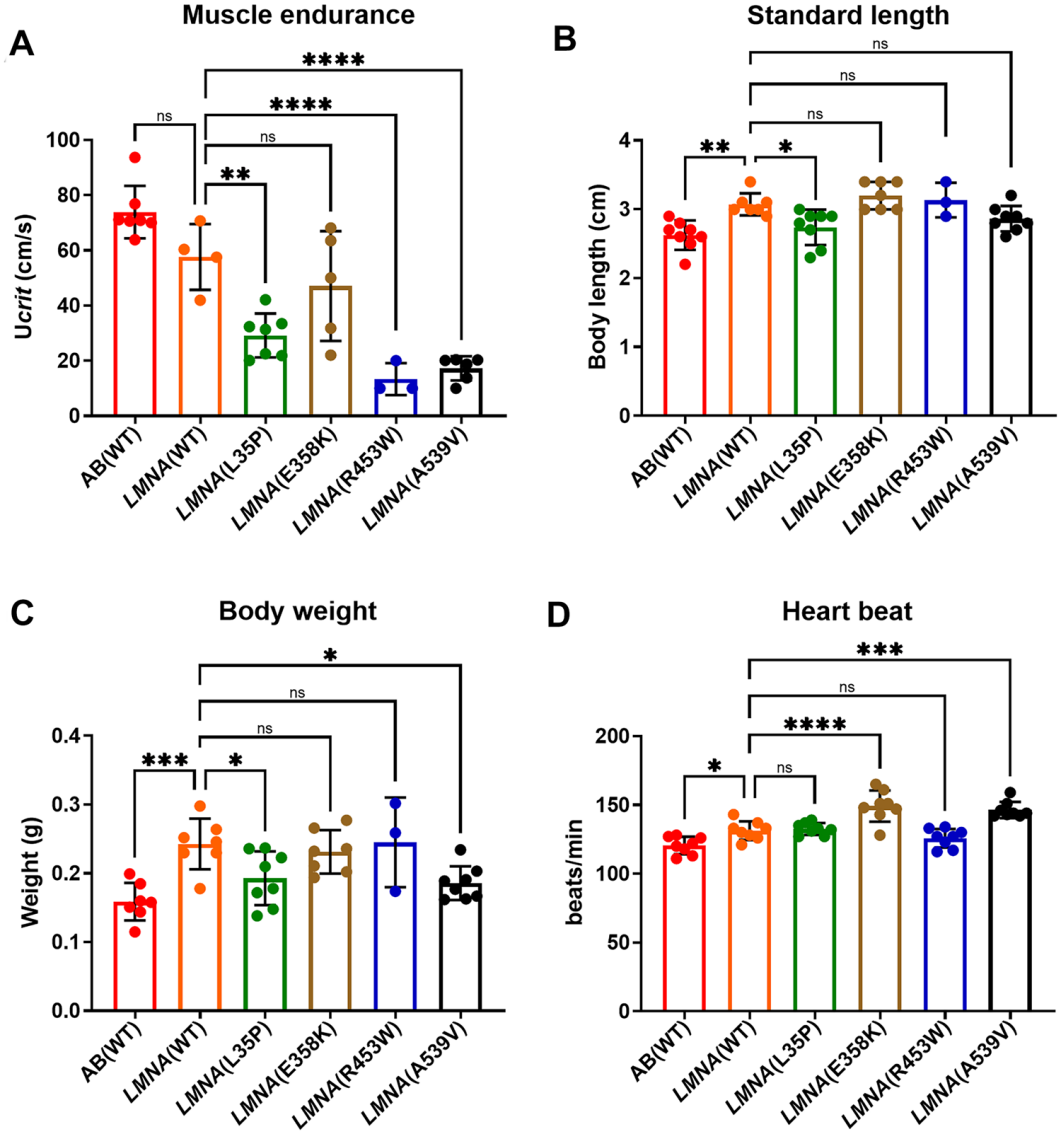
The muscle endurance and heartbeat measurements of F1 adult *LMNA* transgenic zebrafish. (**A**) Muscle endurance was assessed using the critical swim speed (*U*_crit_) measured in a Brett-type swimming tunnel. AB(WT): n = 6; *LMNA*(WT): n = 4; *LMNA*(L35P): n = 7; *LMNA*(E358K): n = 5; *LMNA*(R453W): n = 3; *LMNA*(A539V): n = 6. (**B**) The length of the adult fish were shown. AB(WT): n = 8; *LMNA*(WT): n = 7; *LMNA*(L35P): n = 8; *LMNA*(E358K): n = 7; *LMNA*(R453W): n = 3; *LMNA*(A539V): n = 8. (**C**) body weight of the adult fish were recorded. AB(WT): n = 8; *LMNA*(WT): n = 7; *LMNA*(L35P): n = 8; *LMNA*(E358K): n = 6; *LMNA*(R453W): n = 3; *LMNA*(A539V): n = 8. (**D**) The heartbeat of 4dpf F1 larval fish with different LMNA transgenic variations was measured. AB(WT): n = 8; *LMNA*(WT): n = 8; *LMNA*(L35P): n = 8; *LMNA*(E358K): n = 8; *LMNA*(R453W): n = 8; *LMNA*(A539V): n = 8. The red plot represents AB(WT) fish, the orange plot for *LMNA*(WT) fish, the green plot for *LMNA*(L35P) fish, the brown plot for *LMNA*(E358K) fish, the blue plot for *LMNA*(R453W) fish, and the black plot for *LMNA*(A539V) fish. Each dot represents one fish. Statistical analysis was conducted using ordinary One-way ANOVA with Dunnett’s test. The presented P-values have been appropriately adjusted, and the level of statistical significance is denoted as follows: *for 0.01 < P ≤ 0.05, **for 0.001 < P ≤ 0.01, ***for 0.0001 < P ≤ 0.001, and ****for P ≤ 0.0001.

In order to assess muscle endurance in adult transgenic zebrafish with *LMNA* variants and minimize the influence of learned behavior, we conducted a swimming tunnel test for the F1 adult fish. Notably, *LMNA*(WT) fish did not exhibit significantly different muscle endurance compared to the AB(WT) fish (Fig. 3A). On the other hand, all of the *LMNA* mutant transgenic fish displayed a discernible decrease in muscle endurance. Particularly noteworthy were the findings regarding *LMNA*(L35P), *LMNA*(R453W), and *LMNA*(A539V) fish, which demonstrated a notable reduction in muscle endurance when compared to *LMNA*(WT) fish (Fig. 3A). The lack of statistical significance in *LMNA*(E358K) may be attributed to a larger standard deviation.

Additionally, we measured the body weight and standard length of the 3-month-old F1 fish and observed that the *LMNA* transgenic fish were longer and heavier than the AB(WT), but no difference compared to *LMNA*(WT) fish (Fig. 3B,C). This finding suggests that the reduced muscle endurance was not solely due to smaller fish size. In summary, our results reveal that various variants in *LMNA*, including two novel variants: *LMNA*(L35P) and *LMNA*(A539V), and two pathogenic LMNA variants *LMNA*(E358K) and *LMNA*(R453W) fish exhibit significantly weaker muscle strength compared to *LMNA*(WT) fish, which emphasize the impact of *LMNA* variants on muscle endurance in adult transgenic fish (Table 2).

### Elevated heartbeat in 4 dpf larval *LMNA* mutant transgenic fish

*LMNA* variants have been reported to be associated with EDMD with cardiac involvement, including bradyarrhythmia, tachyarrhythmia and cardiomyopathy. In our study, we examined the heartbeat of 4-dpf F1 transgenic fish larvae carrying either *LMNA*(WT) or *LMNA* mutants. While the heartbeat of *LMNA*(WT) fish show slightly increased of heart rate compared to AB(WT) fish, we observed an elevated heartbeat in *LMNA*(E358K) and *LMNA*(A539V) fish, compared to *LMNA*(WT) fish (Fig. 3D, Table 2). These findings suggest the presence of cardiac abnormalities is associated with the *LMNA* mutants. However, further investigation is necessary to fully understand the underlying mechanisms involved.

### Histopathological examination reveals abnormal muscle development in *LMNA*(L35P), *LMNA*(E358K), *LMNA*(R453W), and *LMNA*(A539V) transgenic fish

**Figure 4:**
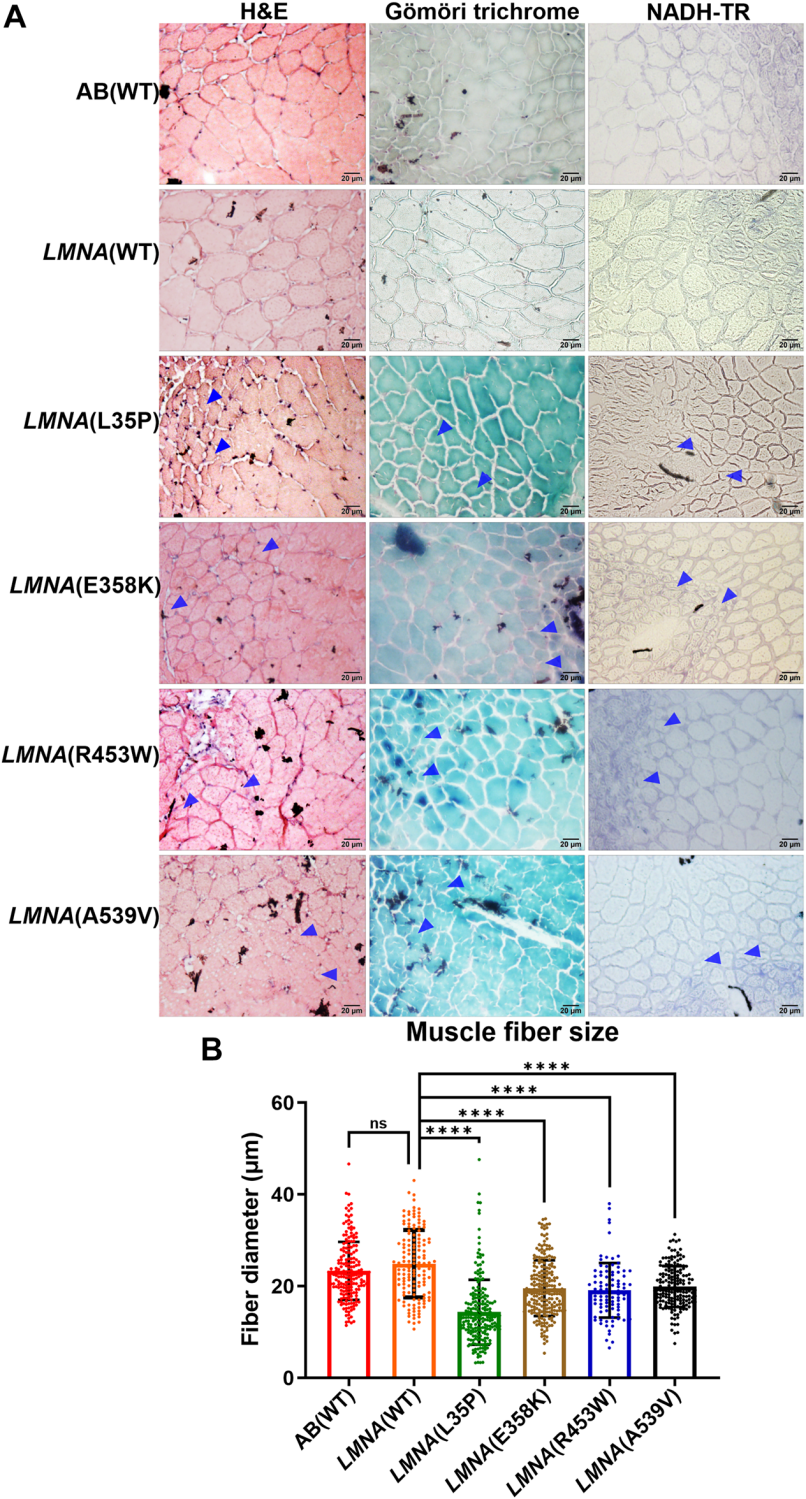
Histopathological analysis of muscle specimens from F1 adult *LMNA* transgenic zebrafish. (**A**) The histopathological characteristics of muscle tissues in F1 adult *LMNA* transgenic zebrafish were conducted by histological staining using H&E stain, Gömöri trichrome stain, and NADH-TR stain. The blue arrowheads indicated the decreased fiber size. The images were captured at a magnification of × 400, and the scale bar represents 20 μm. (**B**) The statistical analysis of muscle fiber size from *LMNA* transgenic zebrafish. The muscle fiber diameter of F1 adult from AB(WT) and *LMNA* transgenic zebrafish was measured. Each dot represents one muscle fiber. AB(WT): n = 198; *LMNA*(WT): n = 141; *LMNA*(L35P): n = 212; *LMNA*(E358K): n = 226; *LMNA*(R453W): n = 92; *LMNA*(A539V): n = 175. The red plot represents AB(WT) fish, the orange plot for *LMNA*(WT) fish, the green plot for *LMNA*(L35P) fish, the brown plot for *LMNA*(E358K) fish, the blue plot for *LMNA*(R453W) fish, and the black plot for *LMNA*(A539V) fish. Statistical analysis was comparing the mean of each column with the mean of no drug control using ordinary One-way ANOVA comparing the mean of each column with the mean of control column (*LMNA*(WT)), and the multiple testing with Dunnett’s test. The presented P-values have been appropriately adjusted, and the level of statistical significance is denoted as follows: *for 0.01 < P ≤ 0.05, **for 0.001 < P ≤ 0.01, ***for 0.0001 < P ≤ 0.001, and ****for P ≤ 0.0001.

To comprehensively investigate the potential link among reduced swimming speed, weaker muscle endurance, and abnormal muscle development in *LMNA* mutant F1 transgenic fish, we performed a detailed histopathological examination of muscles. This examination involved three different stainings including Hematoxylin and eosin (H&E), Gömöri trichrome, and Nicotinamide adenine dehydrogenase (NADH)-TR. Upon conducting the histopathological examinations, our findings revealed significant indications of abnormal muscle development in the *LMNA* mutant transgenic fish, specifically the *LMNA*(L35P), *LMNA*(E358K), *LMNA*(R453W), and *LMNA*(A539V) variants. Notably, we observed diminished fiber size (as indicated by the blue arrowhead) in these transgenic fish (as shown in Fig. 4A). We meticulously measured the dimensions of 92 to 227 muscle fibers to calculate their size, and the resulting statistical analysis is presented in Fig. 4B. Although *LMNA*(WT) has no differences to non-transgenic AB(WT) control, the *LMNA* mutant transgenic fish, particularly those with *LMNA*(L35P), *LMNA*(E358K), *LMNA*(R453W), and *LMNA*(A539V) variants, displayed significantly reduced muscle fiber diameters compared to *LMNA*(WT) group. Table 2 summarizes the pathogenicity of *LMNA* variants, providing an overview of associated phenotypes, swimming speed, muscle endurance, and heartbeat data for the *LMNA* mutations transgenic fish. These histopathological results confirm that the reduced swimming speed and weaker muscle endurance observed in the *LMNA* mutant transgenic fish are indeed associated with the presence of abnormal muscle development.

### Correlation analysis of Lamin A/C overexpression and phenotypic traits

To thoroughly investigate the effects of lamin A/C overexpression on zebrafish phenotypes including changes in swim speed, heart rate, and muscle fiber diameter, we employed correlation matrix analysis using Prism software. This method allowed us to assess the relationship between gene expression levels and observed phenotypes. The correlation coefficient (r) was utilized, ranging from − 1 to + 1, indicating perfect negative correlation, no correlation, and perfect positive correlation, respectively.

Our analysis of zebrafish larval data (refer to Fig. S5A), revealed no significant correlation between lamin A/C expression levels and swim speed. However, we observed correlations between swim speeds at different developmental stages, indicating an intrinsic developmental pattern. In the F1 zebrafish adult data (refer to Fig. S5B), lamin A/C expression levels again showed no significant correlation with swim speed, heart rate, or muscle fiber diameter. There was a modest correlation observed with body length and weight, though these were not statistically significant, with the P-values of 0.367 and 0.293 respectively. Interestingly, swim speeds of day 2, 3 and 5 showed significant correlations with muscle fiber diameter (P-values of 0.046, 0.06 and 0.078 respectively), while heart rate exhibited a negative correlation with swim speed, although not statistically significant.

In conclusion, our findings suggest that overexpression of lamin A/C may not have a directly effect on the examined mutant phenotypes, such as impaired swim speed, increased heart rates, or decreased muscle fiber diameter. Nonetheless, the significant correlations between swim speed and muscle fiber diameter, as well as the observed relationship between swim speed and heart rate, highlight the complex interactions between lamin A/C expression and phenotypic traits in zebrafish. These insights call for further research to uncover the underlying mechanisms. It’s important to note, however, the roles of significant overexpression of the mutant LMNA in these transgenic fish phenotypes cannot be entirely ruled out.

### Elevated swimming speeds in *LMNA*(A539V) larvae with terazosin treatment, *LMNA*(L35P) larvae with L-carnitine treatment and *LMNA*(R453W) larvae with creatine or taurine treatment

At present, there is no definitive treatment for muscular laminopathy, and care options primarily focus on managing symptoms and providing supportive care. In the pursuit of therapeutic drugs for *LMNA*-related muscular laminopathy, we conducted a screening of various chemical compounds on the *LMNA* transgenic fish. The muscle weakness experienced by patients with *LMNA* variants resembles the slower swimming speed and weaker muscle endurance observed in these mutant transgenic fish. Given that an improvement in swimming speed reflects an increase in muscle strength, we administered drugs to the F1 larvae and assessed their swimming speed using DanioVision. The drug treatments were initiated at 28 hpf, and swim speed was examined from 5 to 8 dpf.

**Figure 5:**
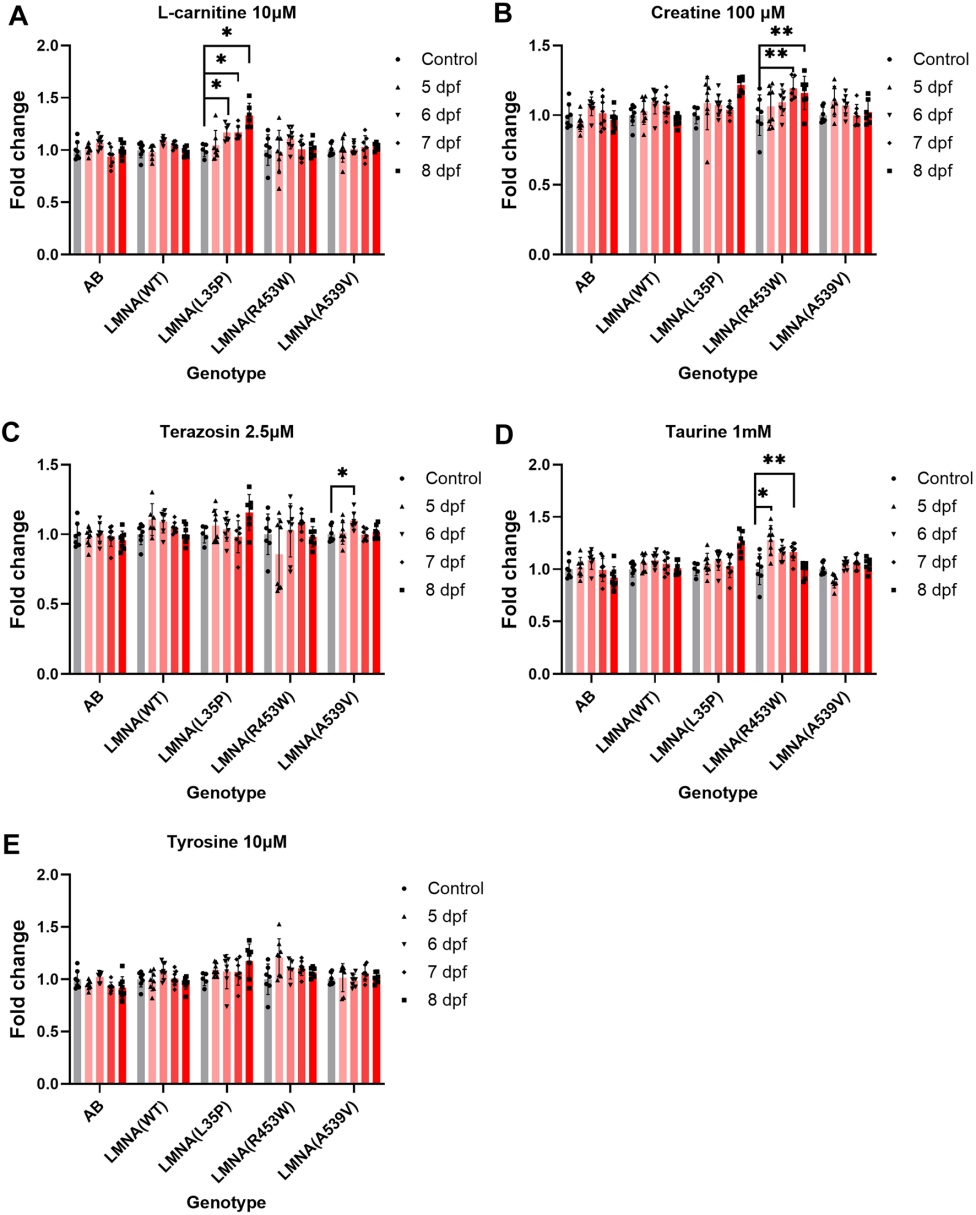
The effects of chemical treatment on F2 heterozygous larvae of *LMNA* transgenic zebrafish. The larvae fish were subjected to chemical treatment, and their swim speed was assessed using DanioVision from 5 to 8 dpf. (**A**) l-carnitine 10 μM, (**B**) Creatine 100 μM, (**C**) Terazosin 2.5 μM, (**D**) Taurine 1 mM, (**E**) Tyrosine 10 μM treatments were administered to AB(WT), *LMNA* (WT), *LMNA*(A539V), *LMNA*(L35P), *LMNA*(R453W) larvae fish. Statistical analysis was conducted using a One-way ANOVA with Dunnett’s test comparing the swim speed of the chemically treated larvae to those with no drug treatment. Each dot represents one fish. AB(WT), *LMNA*(WT), *LMNA*(L35P), *LMNA*(E358K), *LMNA*(R453W), *LMNA*(A539V), n = 8 for each group. The presented P-values have been appropriately adjusted, and the level of statistical significance is expressed as a P-value, where indicated as following: *represents 0.01 < P ≤ 0.05, **represents 0.001 < P ≤ 0.01.

Based on our previous report, we selected five chemical drugs: l-carnitine, creatine, terazosin, taurine, and tyrosine that have shown potential in improving neuromuscular diseases^28^. Since *LMNA*(E358K) which showed a slightly lower muscle endurance trend, we tested AB(WT), *LMNA*(WT), *LMNA*(L35P), *LMNA*(R453W), and *LMNA*(A539V) fish with these five selected drugs (Fig. 5). The comparisons were made within the same drug treatment group to see if there was an improvement in the swimming capacity of the fish with a certain genotype after treatment with drug. l-carnitine treatment significantly increased the swim speed of *LMNA*(L35P) larvae at 6 ~ 8 dpf (Fig. 5A). Moreover, creatine treatment notably enhanced the swim speed of *LMNA*(R453W) larvae at 7 and 8 dpf (Fig. 5B). Intriguingly, terazosin treatment improved the swim speed of *LMNA*(A539V) larvae at 6 dpf (Fig. 5C). Additionally, taurine treatment markedly boosted the swimming speed of *LMNA*(R453W) larvae at 5 and 7 dpf (Fig. 5D), while tyrosine showed no significant effect on the swim speed of any transgenic fish (Fig. 5E). Encouraged by these results, we selected terazosin, l-carnitine, creatine, and taurine for further investigation by administering them to the larvae fish for one month to evaluate their long term effects on muscle endurance.

### Rescue of muscle endurance in *LMNA*(L35P) with L-carnitine treatment and in *LMNA*(R453W) with creatine treatment

**Figure 6:**
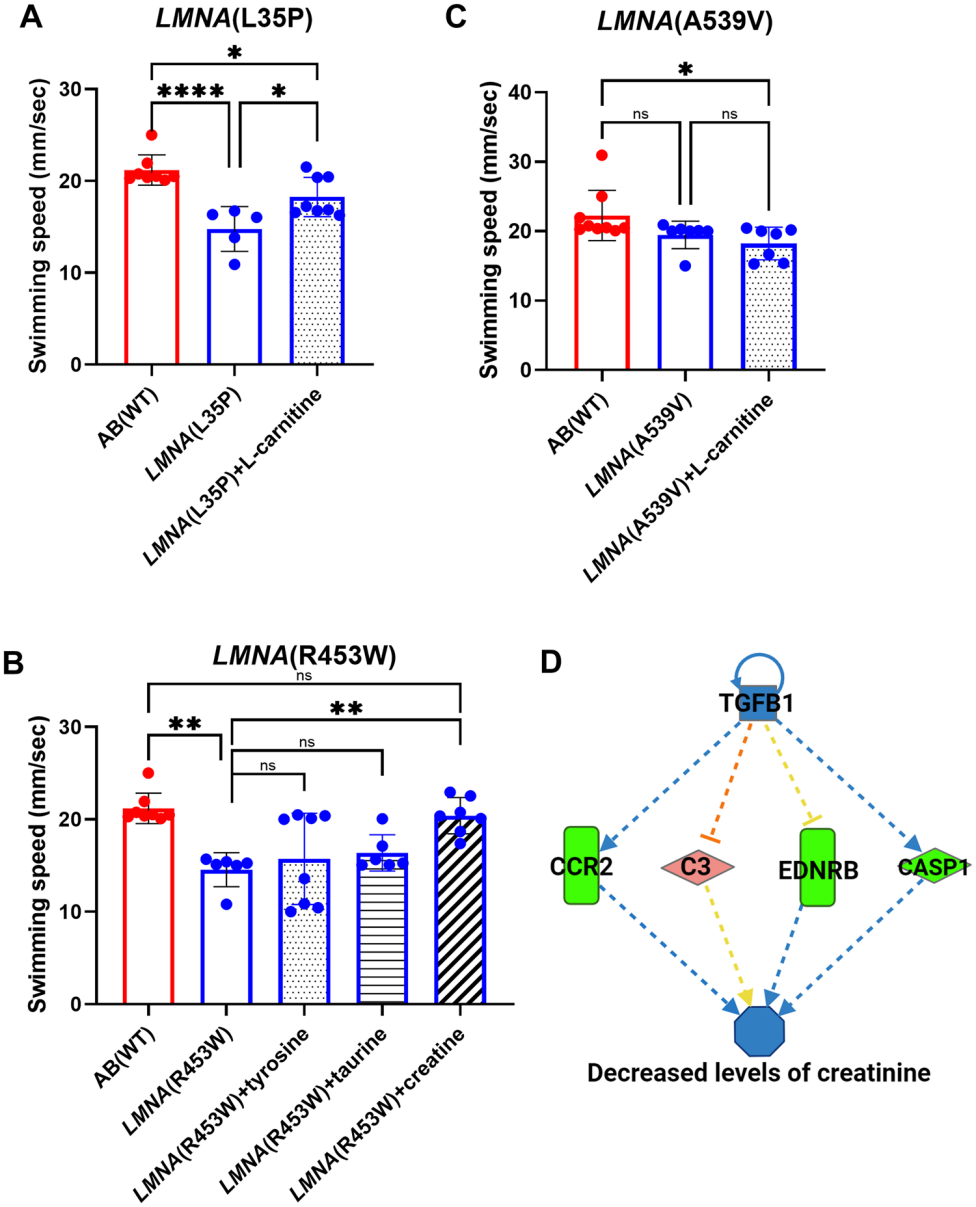
The muscle endurance of 1-month-old F2 heterozygous *LMNA* transgenic zebrafish after treated with chemicals. (**A**) Muscle endurance of AB(WT): n = 8; *LMNA*(L35P): n = 5; *LMNA*(L35P) + l-carnitine: n = 8. (**B**) Muscle endurance of AB(WT): n = 8; *LMNA*(R453W); n = 6; *LMNA*(R453W) + tyrosine: n = 8; *LMNA*(R453W) + taurine: n = 6; *LMNA*(R453W) + creatine: n = 7. (**C**) Muscle endurance of AB(WT): n = 9; *LMNA*(A539V): n = 7, *LMNA*(A539V) + l-carnitine: n = 7. Adult fish were treated with chemical for 1-month. Each dot represents one fish. Statistical analysis was conducted using a One-way ANONVA with Dunnett’s test, comparing the treated groups to either AB(WT) or the no drug treatment group. The presented p-values have been appropriately adjusted, and the level of statistical significance is indicated as following *represents 0.01 < P ≤ 0.05, **represents 0.001 < P ≤ 0.01, and ****represents P ≤ 0.0001. (**D**) Ingenuity Pathway Analysis (IPA) network pathway analysis revealed dysregulated genes in *LMNA*(R453W) mutant transgenic fish, resulting in decreased levels of creatine showing in blue. *TGFB1* transforming growth factor-beta1, *CCR2* C–C chemokine receptor type 2, *C3* complement component 3, *EDNRB* endothelial receptor type B, *CASP1* Caspase 1.

Based on our previous report, drug treatments were initiated at 28 hpf and muscle endurance was evaluated at one month of age^28^, we implemented the same experimental protocol on the three *LMNA* mutant transgenic fish. The transgenic fish were administered with the drug treatments from 28 hpf larvae to 30 dpf adult fish. Notably, we observed a significant improvement in muscle endurance of *LMNA*(L35P) fish after 1 month of l-carnitine treatment (Fig. 6A). In addition, the muscle strength of *LMNA*(R453W) fish was effectively restored through creatine treatment only, while taurine and tyrosine treatments did not produce the similar effects (Fig. 6B). Interestingly, no significant differences in muscle endurance were observed in *LMNA*(A539W) fish by l-carnitine treatment (Fig. 6C). Using Ingenuity Pathway Analysis (IPA) network pathway analysis, we discovered that *LMNA*(R453W) mutant transgenic fish could exhibit decreased levels of creatinine (Fig. 6D), which is a byproduct of creatine. These results provide insights into the effects of creatine treatment on muscle endurance in *LMNA*(R453W) transgenic zebrafish, as well as the dysregulated gene pathways associated with the *LMNA* mutants. These findings highlight the *LMNA* specific mutation nature of muscle endurance reversal achieved through targeted drug treatment.

### Rescue of AMPK and mTOR pathways by creatine treatment in *LMNA*(R453W) fish, but not in L-carnitine treated *LMNA*(L35P) fish

**Figure 7:**
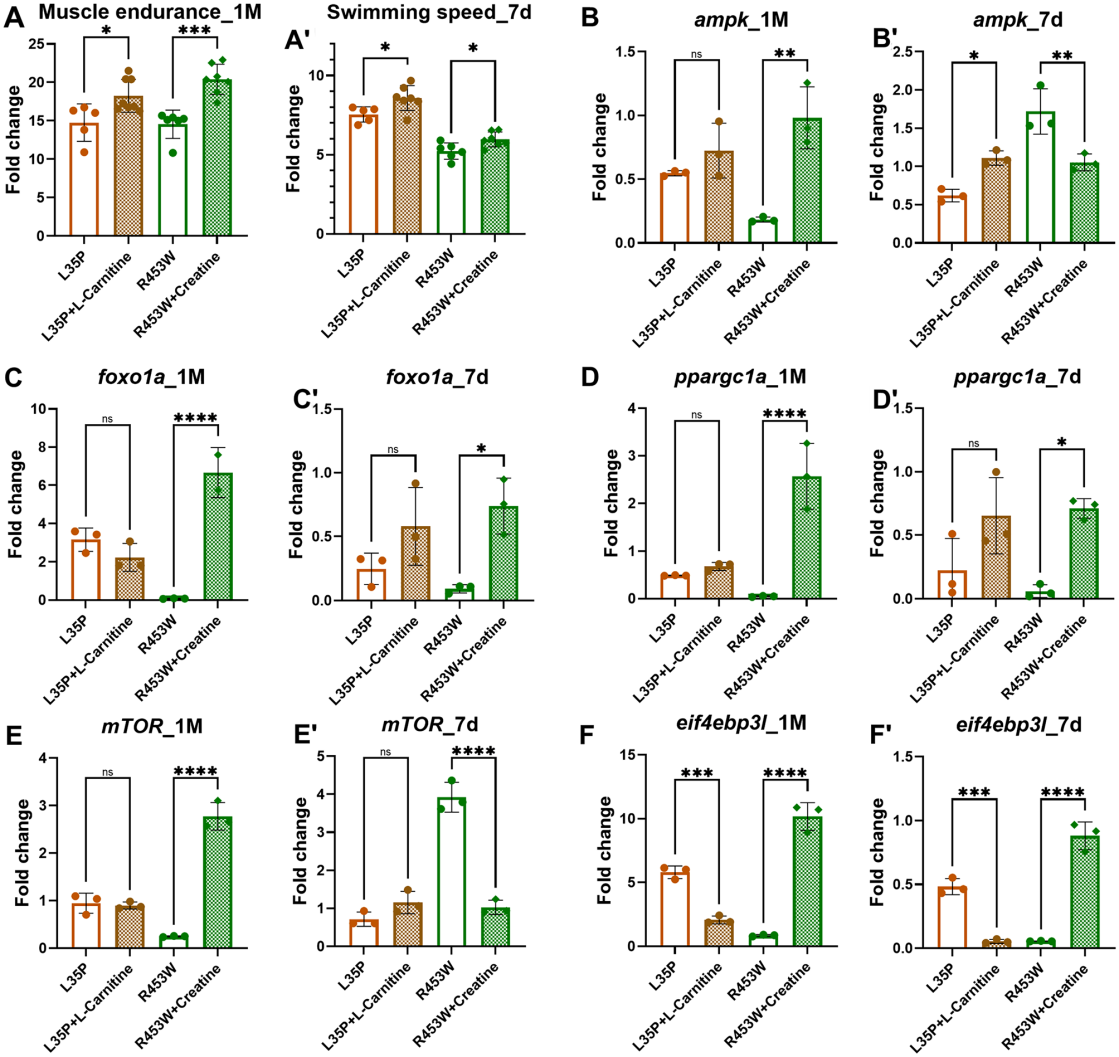
The expression patterns of the AMPK and mTOR pathways in chemical treated 7-day-old larvae and 1-month-old F2 heterozygous *LMNA* transgenic zebrafish. (**A,Aʹ**) Muscle endurance in the 1-month-old *LMNA*(L35P) and *LMNA*(R453W) fish, as well as swimming speed in the 7-day-old larvae with or without chemical treatment. The expression levels of key genes related to the AMPK and mTOR pathways were assessed using qPCR in both 7-day-old larvae and 1-month-old *LMNA* transgenic zebrafish. *LMNA*(L35P): n = 5; *LMNA*(L35P) + l-carnitine: n = 8; *LMNA*(R453W); n = 6; *LMNA*(R453W) + creatine: n = 7. (**B,Bʹ**) Expression of *ampk* gene. (**C,Cʹ**) Expression of *foxo1a* gene. (**D,Dʹ**) Expression of *ppargc1a* gene. (**E,Eʹ**) Expression of *mTOR* gene. (**F,Fʹ**) Expression of *eif4ebp3l* gene. For larvae: n = 20; for adult: n = 10. Statistical analysis was performed using ordinary One-way ANOVA, comparing the expression levels to the no drug treatment group. The presented P-values have been appropriately adjusted, and the level of statistical significance is indicated by the P-value, where denoted as following *represents 0.01 < P ≤ 0.05, **represents 0.001 < P ≤ 0.01, ***represents 0.0001 < P ≤ 0.001, and ****represents P ≤ 0.0001.

Previous studies have reported a decrease in AMPK level in *LMNA* mutant-related muscle disorders^8^. To investigate whether the rescue effect by the drugs is associated with AMPK pathway, we examined the expression levels of AMPK and downstream mTOR pathway markers using RT-qPCR in the *LMNA* mutant transgenic fish with or without treatment. First we confirmed an increase in muscle endurance or in swim speed in *LMNA*(L35P) fish treated with l-carnitine and in *LMNA*(R453W) fish treated with creatine for 1 month (Fig. 7A) or 7 days (Fig. 7Aʹ) compared to those without treatments. Next, we detected that the levels of *ampk* were dramatically elevated in the *LMNA*(R453W) fish treated with creatine for 1 month (Fig. 7B), yet this increase was not detected in 7-dpf larvae (Fig. 7Bʹ), suggesting the long term creatine treatment may enhance *ampk* levels in *LMNA*(R453W) fish for the improvement of muscle endurance and swim speed. On the other hand, a trend of increased *ampk* levels occurred in the l-carnitine-treated 1 month old *LMNA*(L35P) fish but no significant differences (Fig. 7B), yet a significant increase in *ampk* levels was detected in l-carnitine-treated 7-dpf *LMNA*(L35P) larvae (Fig. 7Bʹ).

In explore the impact of AMPK activation on muscle endurance, we conducted further analysis by measuring the expression levels of two key downstream target genes of AMPK, namely *foxo1a* and *ppargc1a*, in *LMNA* mutant transgenic fish, both untreated and treated. *Foxo1a* and *ppargc1a* are well-documented for their crucial roles in muscle growth, development and differentiation^40,41^. Our findings revealed that treatment with creatine significantly elevated the expression of both *foxo1a* and *ppargc1a* in *LMNA*(R453W) fish across different stages, including 1-month-old fish and 7-dpf larvae (Fig. 7C,Cʹ,D,Dʹ). This increase supports the theory that creatine treatment activates the *ampk/foxo1a*/*ppargc1a* pathway in *LMNA*(R453W) fish, contributing to enhanced muscle endurance.

Conversely, treatment with l-carnitine did not yield any significant changes in the expression levels of *foxo1a* and *ppargc1a* in *LMNA*(L35P) fish at similar development stages (Fig. 7C,Cʹ,D,Dʹ). This observation aligns with earlier findings that showed no significant impact of l-carnitine treatment pn AMPK levels in the same fish model (Fig. 7B). Considering *foxo1b* and *ppargc1b* as paralogs to *foxo1a* and *ppargc1a*, respectively, it introduces an interesting avenue for future research to further elucidate the genetic underpinnings of muscle endurance enhancement.

Since mTOR is located downstream of AMPK and mTOR has been well reported to regulate muscle growth and metabolism^42,43^ as well as the eIF4EBP3L as a gatekeeper of mTOR in muscle growth^44^, we next examined the effect of mTOR pathway by measuring the levels of *mTOR* and *eif4ebp3l* in the *LMNA* mutant transgenic fish with or without drug treatment. Intriguingly, similar to the results in *ampk* (Fig. 7B,Bʹ), the levels of *mTOR* expression were also significantly increased in the creatine treated *LMNA*(R453W) fish (Fig. 7E,Eʹ), implicating the possibility of AMPK/mTOR axis in improving muscle endurance in *LMNA*(R453W) fish by creatine treatment. In addition, the levels of *eif4ebp3l* were also increased in *LMNA*(R453W) fish treated with creatine (Fig. 7F,Fʹ), indicating potential involvement of these markers *mTOR* and *eif4ebp3l* in the beneficial effects by creatine treatment. Our findings highlight the potential beneficial effects of activated AMPK and mTOR pathways by creatine treatment specifically in improving muscle endurance and swim speed in the *LMNA*(R453W) fish, yet the beneficial effects by l-carnitine treatment in *LMNA*(L35P) fish did not rely on the AMPK and mTOR pathways, which needs further experiments to identify its potential underlying mechanism. These results suggest that the efficacy of a specific drug treatment in enhancing muscle endurance is specific to a particular mutation, which may help in the development of precision medicine for muscular laminopathy.

### RNAseq analysis reveals motor dysfunction, cardiac abnormalities, and ion flux dysregulation in *LMNA* mutant transgenic fish

**Figure 8:**
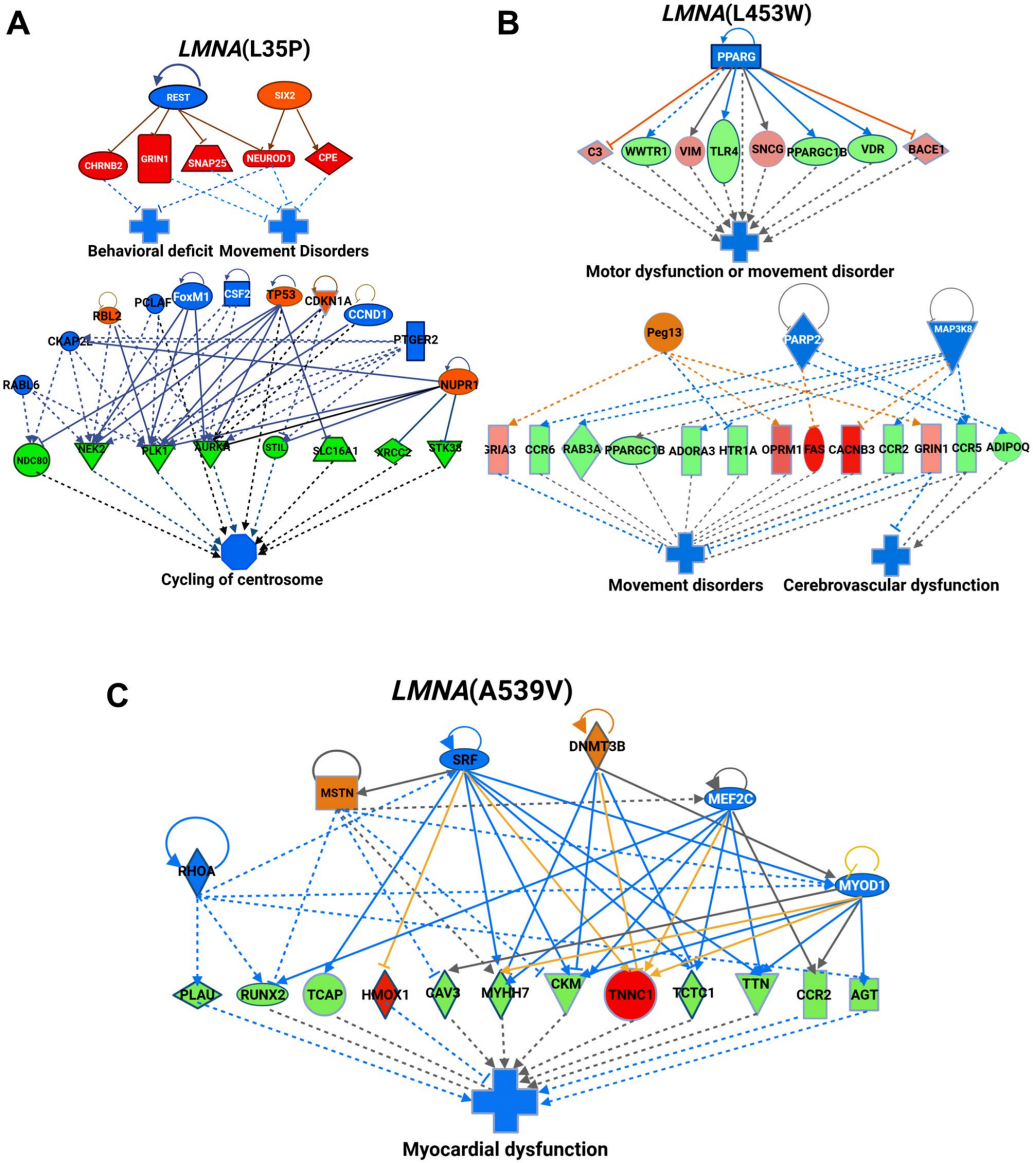
Ingenuity Pathway Analysis (IPA) network pathway analysis uncovers dysregulated genes in *LMNA* mutant transgenic fish. (**A**) F2 *LMNA*(L35P) mutants exhibit dysregulation in genes associated with behavior deficits, movement disorders, and cycling of the centrosome. (**B**) Dysregulation of genes related to movement disorders is observed in F2 *LMNA*(R453W) mutants. (**C**) *LMNA*(A539V) mutants demonstrate dysregulation in genes associated with myocardial dysfunction. These findings obtained through IPA provide insights into the dysregulated pathways and potential molecular mechanisms underlying the observed phenotypic changes in *LMNA* mutant transgenic fish.

In this study, we successfully generated *LMNA* variant transgenic fish and observed distinct effects on their appearance, behavior, and the expression of AMPK- and mTOR-related genes. To attain a comprehensive understanding of the perturbed pathways and genes linked to *LMNA* mutations, an in-depth transcriptomic analysis was conducted utilizing state-of-the-art next-generation sequencing (RNA-seq) technology on the transgenic fish carrying *LMNA* mutations. This meticulous approach yielded a range of significant discoveries. Through the utilization of IPA analysis, it became evident that *LMNA*(L35P), *LMNA*(R453W), and *LMNA*(A539V) variants were closely associated with various movement disorders, motor dysfunction, myocardial issues, and heart failure. The dysregulated genes were color-coded, with green indicating down-regulation and red indicating up-regulation in the *LMNA* mutant context (Fig. 8). Moreover, we successfully identified the upstream regulators governing these genes and the associated disorders, denoted in blue for upregulation and orange for downregulation. In terms of mechanistic elucidation, the *LMNA*(A539V) variant was observed to impede the flow of Ca2 + and the entry of cations, as depicted in Fig. S5A. Correspondingly, the *LMNA*(E358K) variant demonstrated a connection with the inhibition of ion homeostasis and cation transportation, illustrated in Fig. S5B. Additionally, the *LMNA*(R453W) variant was linked to a decline in the mobilization of Ca2+ (Fig. S5C) and a reduction in creatinine levels (Fig. 6D). In summary, the RNAseq results provide valuable insights into the potential underlying pathomechanisms, revealing motor dysfunction, cardiac abnormalities, and dysregulation of ion flux in *LMNA* mutant transgenic fish. These findings contribute to a better understanding of the pathogenesis associated with *LMNA* variants as well as potential interventions against muscular laminopathy.

## Discussion

Our investigation has revealed that transgenic fish harboring *LMNA* mutations exhibit diminished swimming speeds at both larval and adult stages. This reduction in swim speed is consistently observed in both F0 and F1 adult transgenic fish carrying *LMNA*(E358K) and *LMNA*(R453W) variants, mirroring the established pathogenic roles of these variants in individuals with *LMNA*-related CMD and EDMD2. Furthermore, the newly identified *LMNA*(L35P) and *LMNA*(A539V) variants, originating from patients with *LMNA*-related CMD and EDMD, similarly result in decreased swimming speeds in both F0 (refer to Fig. S4D–F) and F1 adult fish (refer to Fig. 2), reinforcing their pathogenic implications.

In addition to the observed alterations in swimming behavior, our findings extend to cardiovascular aspects. Transgenic fish, including those carrying *LMNA*(E358K) and *LMNA*(A539V) variants, exhibit an elevated heartbeat compared to *LMNA*(WT) fish. This cardiac phenotype aligns with the elevated heart rate observed in individuals with both *LMNA*-related CMD and EDMD, providing further evidence of the correlation between the transgenic fish model and the human disease phenotype (refer to Fig. 3D). This comprehensive analysis establishes a parallel between the transgenic fish model and the clinical manifestations seen in patients, offering valuable insights into the pathogenic effects of *LMNA* mutations on both motor and cardiac functions.

The T-maze behavior test is a widely used method to assess spatial learning and memory in vertebrates. In a previous study involving APP and PS1 double transgenic mice, researchers utilized a water maze to investigate swimming speed^45^. In our own experiment, we employed the T-maze to evaluate the swimming speed of adult zebrafish. The fish were trained to locate a specific sponge site that contained food rewards^28^. To enhance their motivation, the fish were deprived of regular food during the testing phase, encouraging them to find the rewarded food as quickly as possible. To eliminate the confounding factors of memory and emotions, we also evaluated muscle endurance in adult fish using a swimming tunnel. By resisting water flow to maintain their position, fish naturally engage their muscles. Different water flow velocities were applied to assess the maximum endurance of the muscles in different transgenic fish carrying *LMNA* mutants which proved weaker muscle endurance in *LMNA* mutants transgenic fish, providing a measure of muscle strength independent of memory and emotional factors.

For larval testing, we assessed swimming speed from 5 to 8 dpf using DanioVision after drug treatment. If the drug treatment increased larval swim speed, we continued the treatments for one month and examined muscle endurance to evaluate the effect of the drug treatment. Our results demonstrated that both *LMNA*(L35P) fish treated with l-carnitine and *LMNA*(R453W) fish treated with creatine consistently exhibited enhancement in larval swimming speed and muscle endurance. Thus, larval swim speed appears to be a valid initial indicator for muscular laminopathy.

Patients with CMD, EDMD, and LGMD exhibit a range of structural and functional muscle abnormalities. In CMD, irregular muscle fiber morphology, including variability in size, fiber splitting, and centrally located nuclei, is common, alongside impaired muscle regeneration, leading to progressive weakness and atrophy. EDMD patients often display disorganized muscle fibers, nuclear blebbing, and altered nuclear envelope proteins like lamin A/C, resulting in spinal rigidity, joint contractures, and cardiac arrhythmias. LGMD presents with both structural issues such as fiber necrosis and fatty infiltration, and functional deficits like reduced muscle strength and mobility.

Our investigation into mutant transgenic fish carrying variants such as *LMNA*(L35P), *LMNA*(E358K), *LMNA*(R453W), and *LMNA*(A539V) has unveiled parallels with CMD, EDMD, and LGMD patients. These fish exhibit slower swim speed, weaker muscle strength, reduced muscle fiber diameters, and, in cases of *LMNA*(A539V) and *LMNA*(E358K), elevated heartbeats. These findings confirm that abnormal muscle development contributes to the observed swimming and muscle endurance issues in these fish. By highlighting these similarities, our research sheds light on the pathophysiology of muscular laminopathy and underscores the importance of zebrafish models in studying these complex disorders.

In our study, we initially focused on mRNA analysis to understand gene expression dynamics, a choice that yielded valuable insights into transcriptional activities. However, the importance of protein-level assessments, especially for deciphering the phenotypic effects of LMNA variants, emerged as a critical oversight. Notably, the availability of well-characterized antibodies for human lamin A/C could facilitate a detailed examination of protein expression, structural integrity, and alterations caused by LMNA mutations. Such protein-level analysis is crucial for understanding how genetic variations in LMNA affect the proteins function and structure, directly linking to the phenotypic diversity seen in laminopathies. This oversight highlights the need for future research to integrate protein assessments, utilizing specific antibodies for lamin A/C, to uncover the quantitative and qualitative impacts of LMNA variants. This approach promises to deepen our understanding of laminopathies, bridging gene expression with protein functionality for a more comprehensive view of these conditions.We further discovered that *LMNA*(L35P) fish showed improvements in swim speed and muscle endurance following l-carnitine treatment. l-carnitine plays a vital role in fat metabolism by facilitating the transport of long-chain fatty acids into the mitochondria matrix for β-oxidation^46^, thereby enhancing energy acquisition. Previous research has shown that l-carnitine supplementation promotes the transition of muscle fibers from type II to type I in obese rats by activating genes encoding molecular regulators such as PGC-1α and PGC-1β^47^. Type I muscle fibers specialize in higher endurance and long-duration contraction^48^, indicating that an increase in the quantity of type I fibers may enhance fish muscle endurance. Additionally, laminopathy is characterized by cytoplasmic aggregation of nuclear envelope proteins, decreased autophagy, and downregulated expression of AMPKα. In a *Drosophila* model, overexpressing PGC-1α, a downstream gene of AMPK, rescued muscle fiber morphology^8^. We also observed an upregulation of *ppargc1a*, the zebrafish gene encoding PGC-1α, in *LMNA*(L35P) fish following l-carnitine treatment, indicating that upregulating *ppargc1a* levels may promote muscle endurance.

Furthermore, our study revealed the specific efficacy of l-carnitine or creatine treatment in *LMNA*(L35P) and *LMNA*(R453W) fish, respectively, suggesting a potential for personalized medicine. Creatine is synthesized in the liver, kidneys, and pancreas, and then transported to skeletal muscle. Creatine kinase facilitates the reversible reaction between creatine and ATP, resulting in the formation of phosphocreatine and ADP, which are stored in muscle^49^. When muscle damage occurs or there is a high-energy demand, creatine kinase breaks down phosphocreatine to produce ATP as a supplemental energy source^50^. Studies have shown that supplementing the diet with creatine in rats with muscle injuries leads to a higher proportion of undamaged muscle fibers compared to those on a normal diet^51^. Treating *LMNA*(R453W) fish with creatine may help reduce muscle damage caused by laminopathy and provide additional energy for skeletal muscle. Our analysis of qPCR data revealed a significant elevation in the AMPK pathway in *LMNA*(R453W) fish following creatine treatment. Therefore, creatine has the potential to reverse the muscular laminopathy and promote muscle function. While creatine is commonly associated with athletic performance benefits, our study specifically emphasizes its efficacy for *LMNA*(R453W) fish, and l-carnitine‘s effectiveness for *LMNA*(L35P) fish aligns with our broader objective of personalized medicine tailored to different LMNA variants.

Muscle biopsies from laminopathy patients have shown decreased levels of AMPKα and increased levels of mTOR. This imbalance leads to a decrease in the expression of AMPK downstream genes, including PGC1-α and FOXO, ultimately resulting in the downregulation of 4E-BP and contributing to muscular laminopathy^8^. We conducted qPCR analysis of the aforementioned genes, *foxo1a*, *ppargc1a*, and *eif4ebp3l*, and confirmed their expressions are associated with the improved muscle endurance and swim speed with the drug treatments in the *LMNA* transgenic zebrafish. These laminopathy biomarkers may also serve as critical targets for therapeutic interventions.

Rapamycin, an mTOR inhibitor, has been shown to improve muscle defects in a fly model of laminopathy^8^. Whether rapamycin has any effects on those *LMNA* variants transgenic fish awaits for further investigation. Given our previous findings of mTOR upregulation in *LMNA*(A539V), inhibiting mTOR with rapamycin in *LMNA*(A539V) fish may potentially rescue muscle strength. In summary, we identify dysregulated pathways and genes in each transgenic fish carrying *LMNA* variants and disclose their influence on physiological systems. Our research may contribute to the development of precise personalized medicine for muscular laminopathy.

In conclusion, our study on zebrafish models revealed that *LMNA* mutations cause abnormal phenotypes, premature death, decreased fiber size, and muscle dysfunction, reflecting the clinical manifestations of muscular laminopathy. Additionally, it proves the pathogenicity of five LMNA protein variants, including L35P, A539V, W520G, E358K, and R453W, observed in this study. We observed abnormal phenotypes in adult zebrafish carrying LMNA protein variants, such as tail and head abnormalities, crooked bodies, and reduced survival rates. Larvae of the transgenic fish showed slower swim speed. Importantly, we identified the therapeutic potential of l-carnitine and creatine in rescuing muscle endurance in specific *LMNA* mutant zebrafish models. l-carnitine treatment improved muscle endurance in *LMNA*(L35P) fish, while creatine treatment reversed muscle endurance in *LMNA*(R453W) fish implying the involvement of AMPK and mTOR pathways. These findings provide valuable insights for future treatments of *LMNA*-related muscular laminopathy in humans, shedding light on potential therapeutic strategies for these challenging orphan diseases.

### Supplementary Information

Supplementary Information.

## Data Availability

The NGS datasets generated and analyzed during the current study are available in the NCBI Gene Expression Omnibus (GEO) repository, under accession code GSE242251 https://www.ncbi.nlm.nih.gov/geo/query/acc.cgi?acc=GSE242251.

## References

[1] Capell BC, Collins FS (2006). Human laminopathies: Nuclei gone genetically awry. Nat. Rev. Genet..

[2] Tenga R, Medalia O (2020). Structure and unique mechanical aspects of nuclear lamin filaments. Curr. Opin. Struct. Biol..

[3] Earle AJ (2020). Mutant lamins cause nuclear envelope rupture and DNA damage in skeletal muscle cells. Nat. Mater..

[4] Crasto S, My I, Di Pasquale E (2020). The broad spectrum of LMNA cardiac diseases: From molecular mechanisms to clinical phenotype. Front. Physiol..

[5] Shin JY, Worman HJ (2022). Molecular pathology of laminopathies. Annu. Rev. Pathol..

[6] Owens DJ (2020). Lamin-related congenital muscular dystrophy alters mechanical signaling and skeletal muscle growth. Int. J. Mol. Sci..

[7] Xiong L (2020). Linking skeletal muscle aging with osteoporosis by lamin A/C deficiency. PLoS Biol..

[8] Chandran S (2019). Suppression of myopathic lamin mutations by muscle-specific activation of AMPK and modulation of downstream signaling. Hum. Mol. Genet..

[9] Ivorra C (2006). A mechanism of AP-1 suppression through interaction of c-Fos with lamin A/C. Genes Dev..

[10] Hamalainen RH (2019). Defects in mtDNA replication challenge nuclear genome stability through nucleotide depletion and provide a unifying mechanism for mouse progerias. Nat. Metab..

[11] Chemla JC, Kanter RJ, Carboni MP, Smith EC (2010). Two children with “dropped head” syndrome due to lamin A/C mutations. Muscle Nerve.

[12] Cesar S (2023). LMNA-related muscular dystrophy: Identification of variants in alternative genes and personalized clinical translation. Front. Genet..

[13] Liang WC (2007). Novel LMNA mutation in a Taiwanese family with autosomal dominant Emery–Dreifuss muscular dystrophy. J. Formos Med. Assoc..

[14] Mercuri E (2004). Extreme variability of phenotype in patients with an identical missense mutation in the lamin A/C gene: From congenital onset with severe phenotype to milder classic Emery–Dreifuss variant. Arch. Neurol..

[15] Liang WC (2017). Comprehensive target capture/next-generation sequencing as a second-tier diagnostic approach for congenital muscular dystrophy in Taiwan. PLoS ONE.

[16] Bonne G (1999). Mutations in the gene encoding lamin A/C cause autosomal dominant Emery–Dreifuss muscular dystrophy. Nat. Genet..

[17] Liang WC (2020). Clinical, pathological, imaging, and genetic characterization in a Taiwanese cohort with limb-girdle muscular dystrophy. Orphanet J. Rare Dis..

[18] Heller SA, Shih R, Kalra R, Kang PB (2020). Emery–Dreifuss muscular dystrophy. Muscle Nerve.

[19] Corpet F (1988). Multiple sequence alignment with hierarchical clustering. Nucleic Acids Res..

[20] Keller ET, Murtha JM (2004). The use of mature zebrafish (*Danio rerio*) as a model for human aging and disease. Comp. Biochem. Physiol. C Toxicol. Pharmacol..

[21] Oliveira NAS, Pinho BR, Oliveira JMA (2023). Swimming against ALS: How to model disease in zebrafish for pathophysiological and behavioral studies. Neurosci. Biobehav. Rev..

[22] Fazio M, Ablain J, Chuan Y, Langenau DM, Zon LI (2020). Zebrafish patient avatars in cancer biology and precision cancer therapy. Nat. Rev. Cancer.

[23] Chahardehi AM, Hosseini Y, Mahdavi SM, Naseh I (2023). Zebrafish, a biological model for pharmaceutical research for the management of anxiety. Mol. Biol. Rep..

[24] Chia K, Klingseisen A, Sieger D, Priller J (2022). Zebrafish as a model organism for neurodegenerative disease. Front. Mol. Neurosci..

[25] Li M, Hromowyk KJ, Amacher SL, Currie PD (2017). Muscular dystrophy modeling in zebrafish. Methods Cell Biol..

[26] Bassett DI, Currie PD (2003). The zebrafish as a model for muscular dystrophy and congenital myopathy. Hum. Mol. Genet..

[27] Lin YY (2012). Muscle diseases in the zebrafish. Neuromuscul. Disord..

[28] Hsu PJ (2021). L-Carnitine ameliorates congenital myopathy in a tropomyosin 3 de novo mutation transgenic zebrafish. J. Biomed. Sci..

[29] Brown CA (2001). Novel and recurrent mutations in lamin A/C in patients with Emery–Dreifuss muscular dystrophy. Am. J. Med. Genet..

[30] Lu JW (2013). Liver-specific expressions of HBx and src in the p53 mutant trigger hepatocarcinogenesis in zebrafish. PLoS ONE.

[31] Chou YT (2018). Identification of a noncanonical function for ribose-5-phosphate isomerase A promotes colorectal cancer formation by stabilizing and activating beta-catenin via a novel C-terminal domain. PLoS Biol..

[32] Shao YT, Chuang SY, Chang HY, Tseng YC, Shao KT (2018). Largescale mullet (*Planiliza macrolepis*) can recover from thermal pollution-induced malformations. PLoS ONE.

[33] Sztal TE (2018). Testing of therapies in a novel nebulin nemaline myopathy model demonstrate a lack of efficacy. Acta Neuropathol. Commun..

[34] Boyd PJ (2017). Bioenergetic status modulates motor neuron vulnerability and pathogenesis in a zebrafish model of spinal muscular atrophy. PLoS Genet..

[35] Sztal TE, Currie PD, Bryson-Richardson RJ (2017). Analysis of RNA expression in adult zebrafish skeletal muscle. Methods Mol. Biol..

[36] Bertrand AT (2012). DelK32-lamin A/C has abnormal location and induces incomplete tissue maturation and severe metabolic defects leading to premature death. Hum. Mol. Genet..

[37] Toulany N (2023). Uncovering developmental time and tempo using deep learning. Nat. Methods.

[38] Hasegawa Y (2015). Variability of gene expression identifies transcriptional regulators of early human embryonic development. PLoS Genet..

[39] Rivera HE (2021). A framework for understanding gene expression plasticity and its influence on stress tolerance. Mol. Ecol..

[40] Ma M (2022). PPARGC1A is a moderator of skeletal muscle development regulated by miR-193b-3p. Int. J. Mol. Sci..

[41] Xu M, Chen X, Chen D, Yu B, Huang Z (2017). FoxO1: A novel insight into its molecular mechanisms in the regulation of skeletal muscle differentiation and fiber type specification. Oncotarget.

[42] Gol’berg ND, Druzhevskaia AM, Rogozkin VA, Akhmetov II (2014). The role of mTOR in the regulation of skeletal muscle metabolism. Fiziol Cheloveka.

[43] You JS, Anderson GB, Dooley MS, Hornberger TA (2015). The role of mTOR signaling in the regulation of protein synthesis and muscle mass during immobilization in mice. Dis. Model. Mech..

[44] Yogev O, Williams VC, Hinits Y, Hughes SM (2013). eIF4EBP3L acts as a gatekeeper of TORC1 in activity-dependent muscle growth by specifically regulating Mef2ca translational initiation. PLoS Biol..

[45] Liu L (2002). The effects of long-term treatment with metrifonate, a cholinesterase inhibitor, on cholinergic activity, amyloid pathology, and cognitive function in APP and PS1 doubly transgenic mice. Exp. Neurol..

[46] Gnoni A, Longo S, Gnoni GV, Giudetti AM (2020). Carnitine in human muscle bioenergetics: Can carnitine supplementation improve physical exercise?. Molecules.

[47] Kaup D (2018). The carnitine status does not affect the contractile and metabolic phenotype of skeletal muscle in pigs. Nutr. Metab..

[48] Wilson JM (2012). The effects of endurance, strength, and power training on muscle fiber type shifting. J. Strength Cond. Res..

[49] McLeish MJ, Kenyon GL (2005). Relating structure to mechanism in creatine kinase. Crit. Rev. Biochem. Mol. Biol..

[50] Kreider RB, Stout JR (2021). Creatine in health and disease. Nutrients.

[51] Cooke MB, Rybalka E, Stathis CG, Hayes A (2018). Myoprotective potential of creatine is greater than whey protein after chemically-induced damage in rat skeletal muscle. Nutrients.

