# Supplementary material for: Creatine and l-carnitine attenuate muscular laminopathy in the LMNA mutation transgenic zebrafish
Source: Sci Rep. 2024 Jun 4;14:12826. doi: 10.1038/s41598-024-63711-7 (PMC11150447; doi:10.1038/s41598-024-63711-7)
Supplement: Supplementary file 1 — Supplementary Information. [file 41598_2024_63711_MOESM1_ESM.pdf]

Table S1. The primer information for generating pME-*LMNA*, LR reaction confirmation and qPCR analysis

Primer name	ID	Sequence
attB1-LMNA-F	NM_170707.4	GGGGACAAGTTTGTACAAAAAAGCAGGCTATGGAGACCCCGTCCCAGC
attB1-LMNA-R		GGGGACCACTTTGTACAAGAAAGCTGGGTTTACATGATGCTGCAGTTCTGGGG
MLC2-F2	NM_001406916.1	CCATCACTTTCCCCCTACCT
LMNA-R	NM_170707.4	TTACATGATGCTGCAGTTCTGGGG
LMNA-F		ATGGAGACCCCGTCCCAGC
p3E-polyAR		CCCCCTGAACCTGAAACATA
actin-F	NM_131031.2	CTCCATCATGAAGTGCGACGT
actin-R		CAGACGGAGTATTTGCGCTCA
LMNA-F	NM_170707.4	AAAGCGCGCAATACCAAGAA
LMNA-R		CGCAGCATCTCATCCTGAAG
ampk-F	NM_001110286.1	AATCCCCAGAAGTCCAAGCA
ampk-R		TAGGTCCTGCTGTCCACTTG
ppargc1α-F	NM_001135136.1	CTGCCTTGGTTGGTGAAGAC
ppargc1α-R		CCAGCAAGTTGGCCTCATTT
foxo1a-F	NM_001077257.2	CCTGATCACCAAAGCCATCG
foxo1a-R		AGTCCCCTCATTCTGCACTC
mTOR-F	NM_001077211.3	GAGATGGAGGAGATCACGCA
mTOR-R		GAACAGGTGCAGCTCCAAAA
eif4ebp3l-F	NM_199683.1	GTCCTGCACCTCAAAGACTG
eif4ebp3l-R		AGGGTGGAGTGAAGGAATGG

Table S2. The primer information for site-directed mutagenesis

Primer name	Sequence
LMNA T104C-F	CATTGAGCTCCTGCGGGTCCTCCTTCTCC
LMNA T104C-R	GGAGAAGGAGGACCCGCAGGAGCTCAATG
LMNA G1072A-F	GAAGCTCCTGGTACTTGTCCAGCTGCTGCTG
LMNA G1072A-R	CAGCAGCAGCTGGACAAGTACCAGGAGCTTC
LMNA C1357T-F	TTGTTGCGCAGCCAGACAAACTTGCCCTCC
LMNA C1357T-R	GGAGGGCAAGTTTGTCTGGCTGCGCAACAA
LMNA T1558G-F	TCCCGCAGCCCCCGGTGTTCTGTGC
LMNA T1558G-R	GCACAGAACACCGGGGGCTGCGGGA
LMNA C1616T-F	CCAGCTTGCGCATGACCACTTCTTCCCCA
LMNA C1616T-R	TGGGGAAGAAGTGGTCATGCGCAAGCTGG

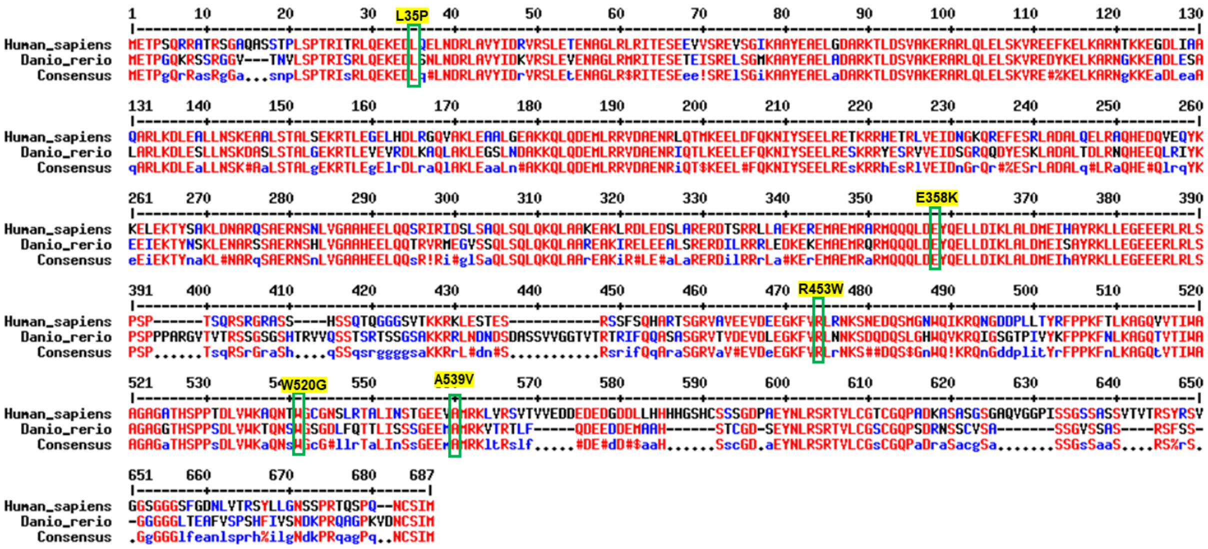

Figure S1. Conservation between human and zebrafish LMNA amino acid sequences. Comparative analysis of the LMNA amino acid sequence from human (ID: NP_733821.1) and zebrafish (ID: NP_694503.1) confirmed that the affected amino acids in our study are indeed conserved in the zebrafish lamin A/C protein. Sequence alignment were done by using Multiple sequence alignment by Florence Corpet (http://multalin.toulouse.inra.fr/multalin/multalin.html).

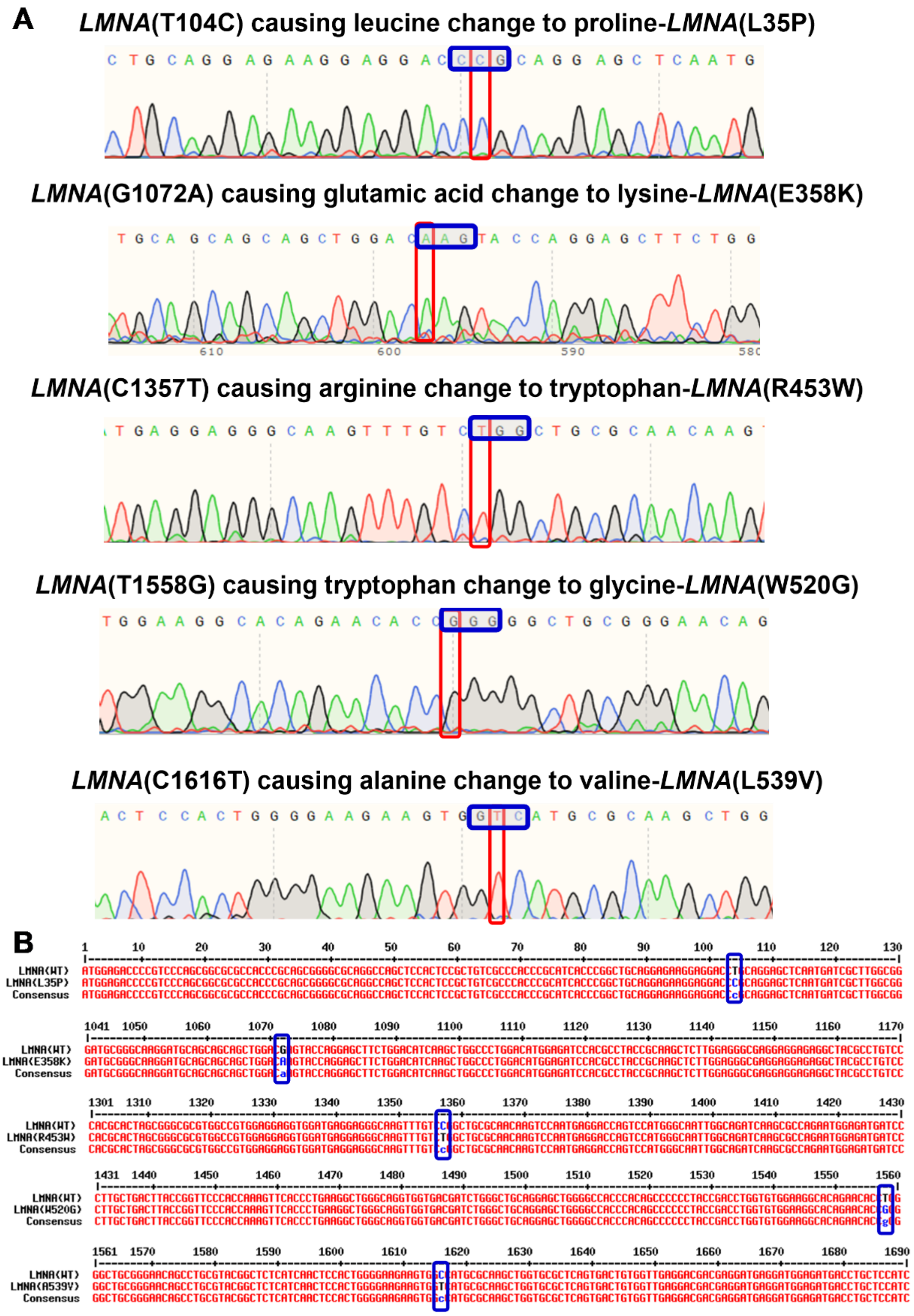

Figure S2. Validation of the *LMNA* mutant transgenic zebrafish sequences. (A) Sequencing analysis confirms the mutation site in each of the *LMNA* mutant transgenic zebrafish. (B) Comparative analysis of the *LMNA* mutant transgenic zebrafish sequence with the wild type *LMNA* (ID: NM_170707.4).

Figure S3

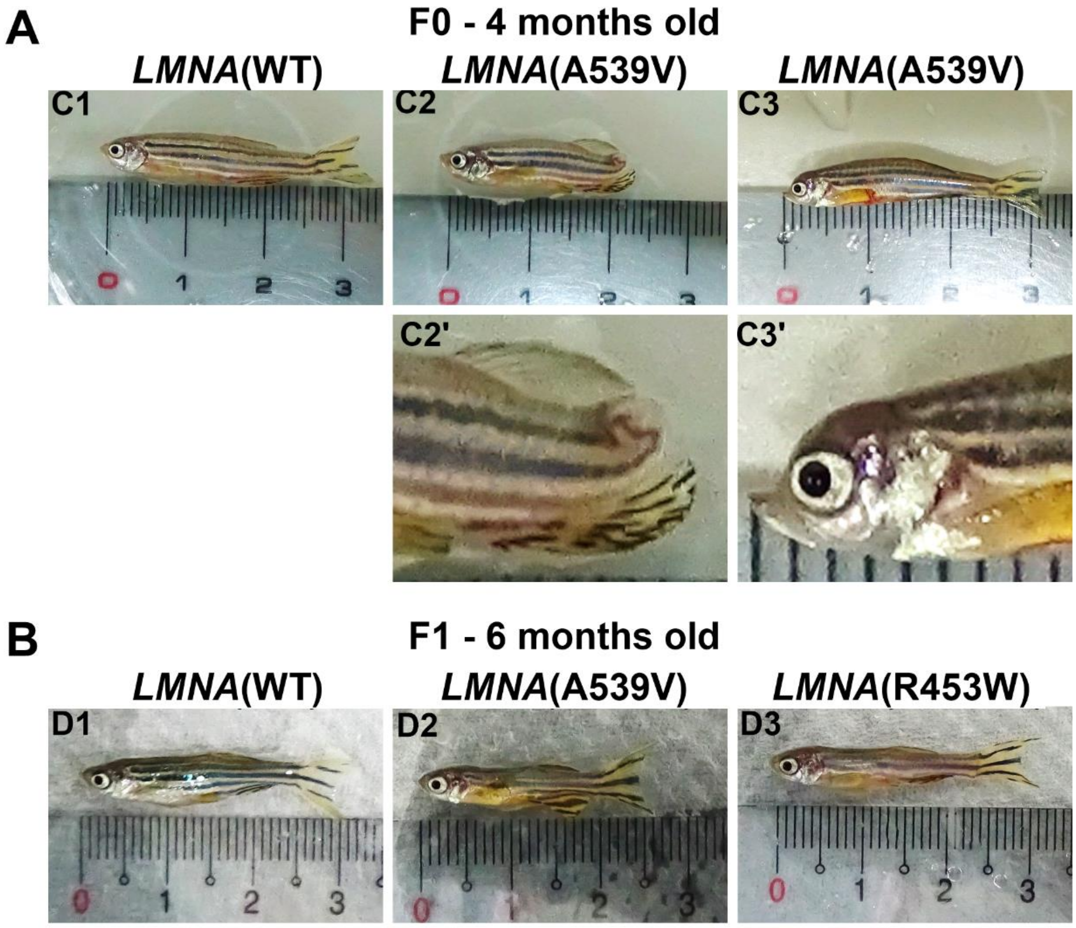

Figure S3. Abnormal phenotypic features in the *LMNA* transgenic zebrafish. (A) Phenotypic appearances of 4-month-old F0 adult *LMNA* transgenic zebrafish. (B) Phenotypic appearances of 6-month-old F1 adult *LMNA* transgenic zebrafish.

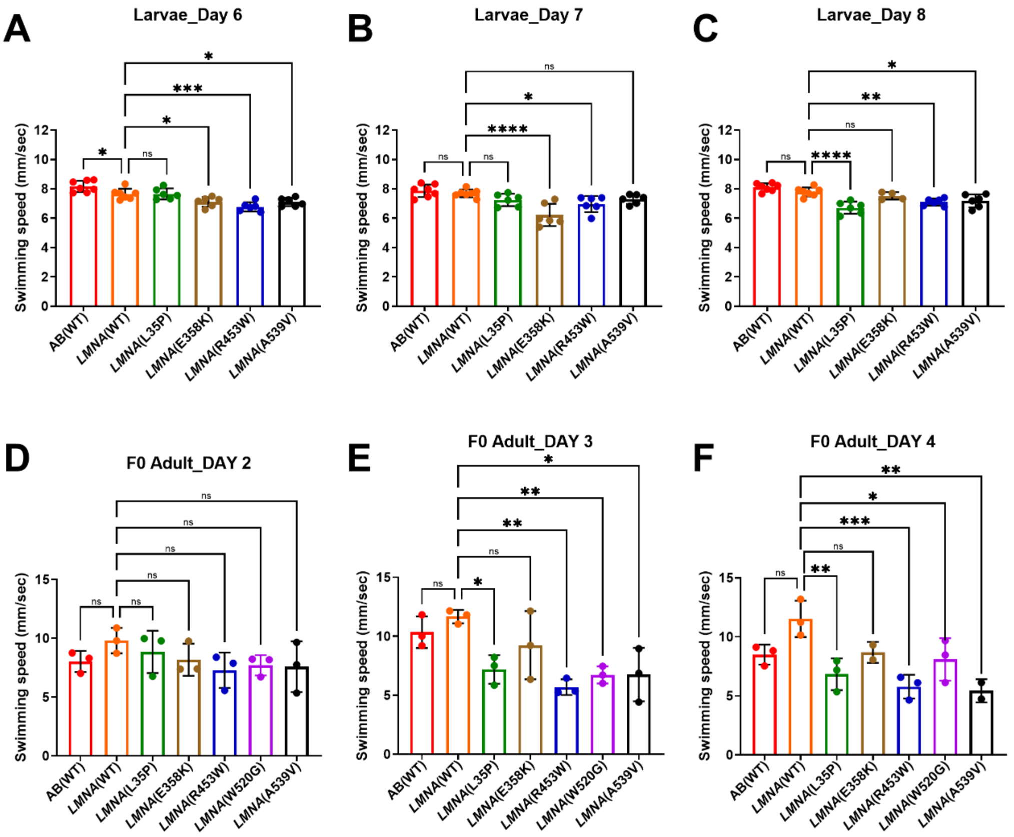

Figure S4. Swimming speed analysis of F1 larvae and F0 adult in the *LMNA* transgenic zebrafish. (A-C) Swimming speed analysis of *LMNA* transgenic zebrafish larvae at 6-8 days post-fertilization (dpf). Larval fish swimming velocity was measured using DanioVision. (D-F) Swimming velocity analysis of F0 adult fish using T-maze behavior. The red plot represents AB(WT) fish, orange plot represents *LMNA*(WT) fish, green plot represents *LMNA*(L35P) fish, brown plot for *LMNA*(E358K) fish, blue plot for *LMNA*(R453W) fish, purple plot for *LMNA*(W520G) fish, and black plot for *LMNA*(A539V) fish. Each dot represents one fish. Statistical analysis was performed using ordinary One-way ANOVA, and corrected for multiple comparisons using Dunnett's test. The presented p-values have been appropriately adjusted, and the level of statistical significance is indicated by the following notation: *: $0.01 < P \leq 0.05$; **: $0.001 < P \leq 0.01$; ***: $0.0001 < P \leq 0.001$; ****: $P \leq 0.0001$.

Figure S5

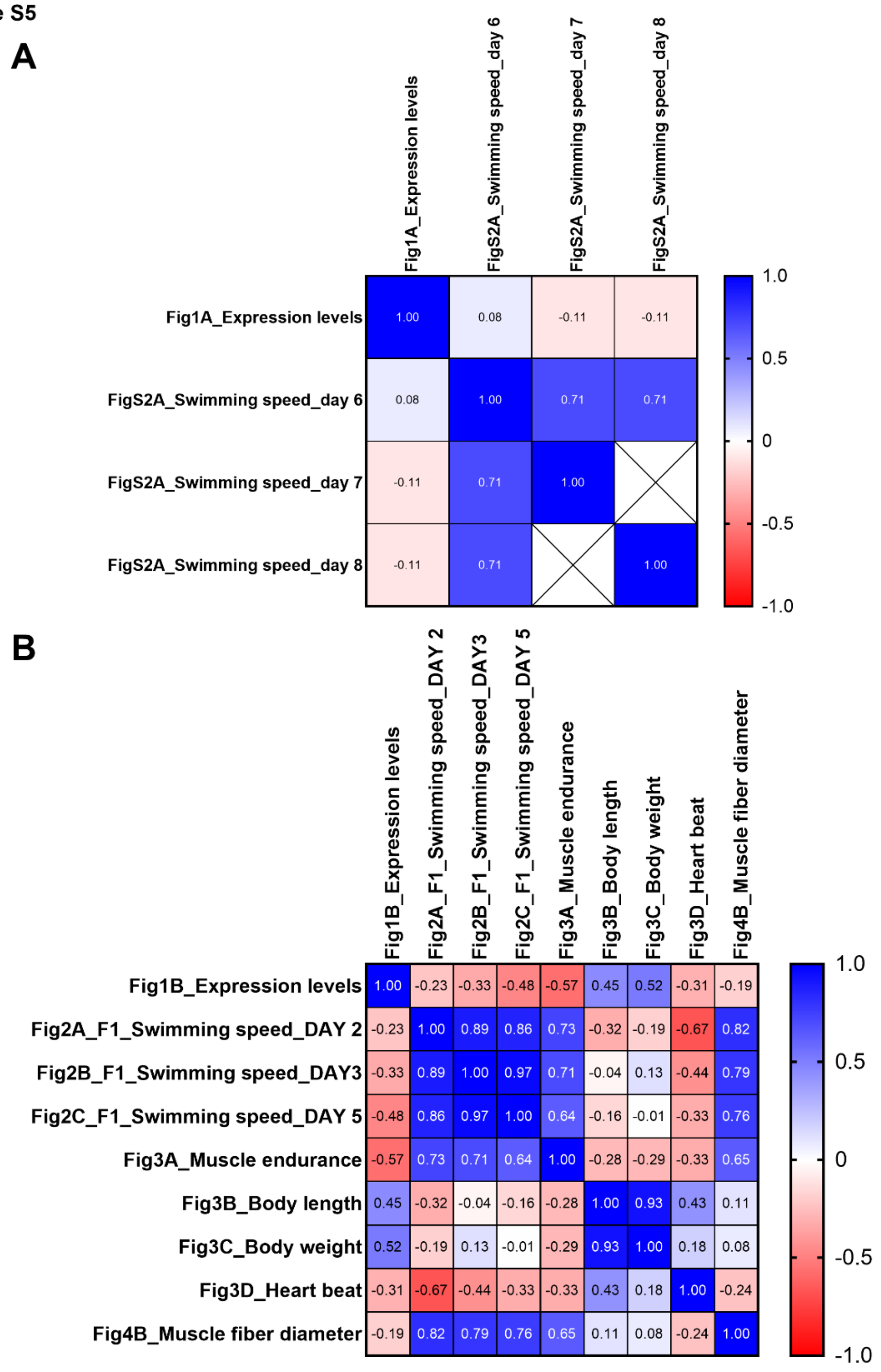

Figure S5. Correlation analysis of larvae and adult in the LMNA transgenic zebrafish. (A) Correlation analysis of expression levels and swim speed of LMNA transgenic zebrafish larvae. (B) Correlation analysis of expression levels along with data on swim speed, muscle endurance, heart rates, and muscle fiber diameter of LMNA transgenic zebrafish adult.

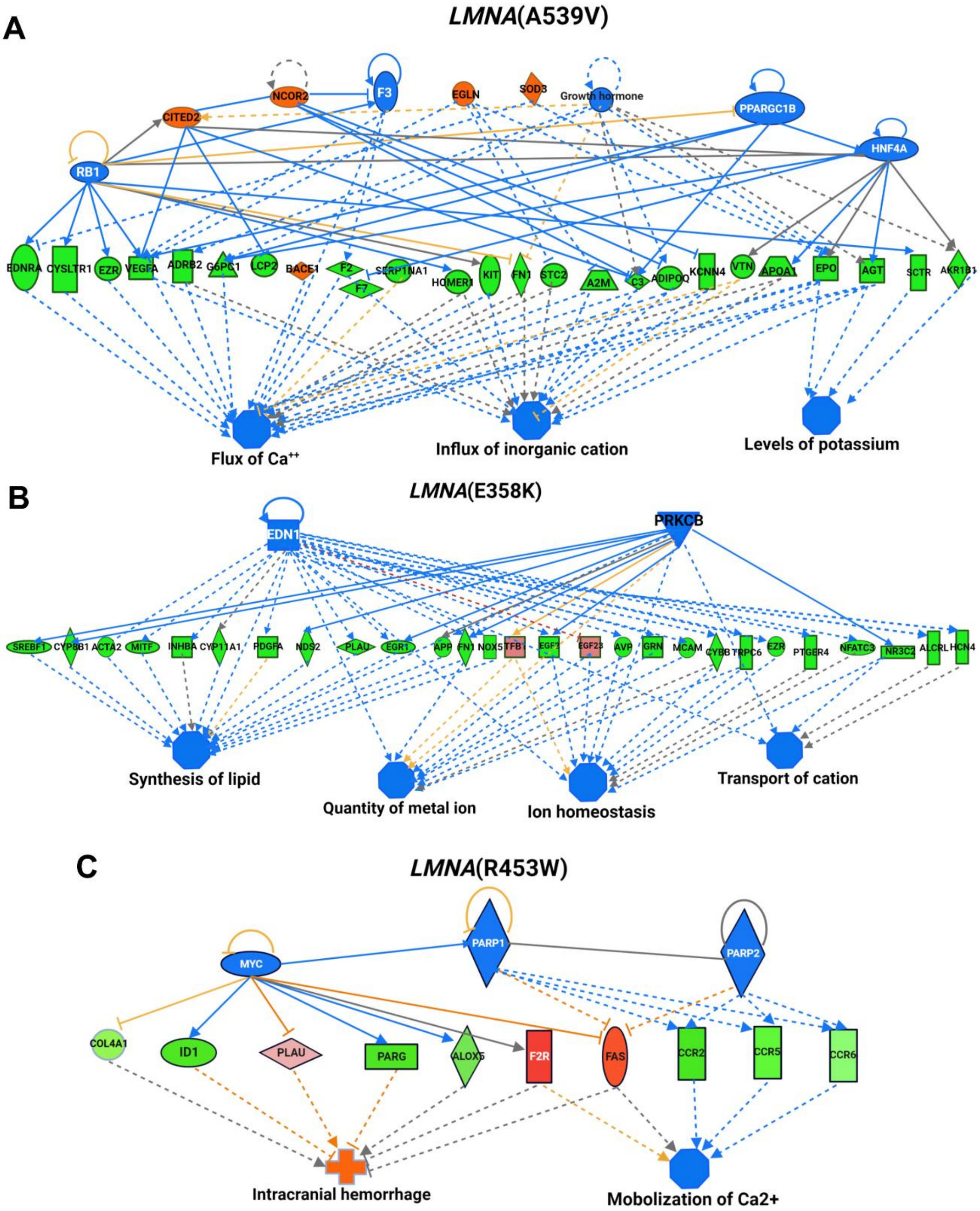

Figure S6. Ingenuity Pathway Analysis network pathway analysis reveals dysregulated genes in *LMNA*(A539V), *LMNA*(E358K) and *LMNA*(R453W) transgenic fish related to ion transport defects. (A) Dysregulated genes associated with ion transport defects are found in *LMNA*(A539V) transgenic fish. (B) Dysregulated genes associated with ion transport defects are observed in *LMNA*(E358K) transgenic fish. (C) Dysregulated genes associated with ion transport defects are observed in *LMNA*(R453W) transgenic fish. These findings obtained through IPA enlighten the dysregulated molecular pathways underlying ion transport defects.
